# A method to identify trace sulfated IgG N-glycans as biomarkers for rheumatoid arthritis

**DOI:** 10.1038/s41467-017-00662-w

**Published:** 2017-09-20

**Authors:** Jing-Rong Wang, Wei-Na Gao, Rudolf Grimm, Shibo Jiang, Yong Liang, Hua Ye, Zhan-Guo Li, Lee-Fong Yau, Hao Huang, Ju Liu, Min Jiang, Qiong Meng, Tian-Tian Tong, Hai-Hui Huang, Stephanie Lee, Xing Zeng, Liang Liu, Zhi-Hong Jiang

**Affiliations:** 1 State Key Laboratory of Quality Research in Chinese Medicine, Macau University of Science and Technology, Avenida Wai Long, Taipa, Macau China; 2 Macau Institute for Applied Research in Medicine and Health, Macau University of Science and Technology, Avenida Wai Long, Taipa, Macau China; 3 Agilent Technologies, 5301 Stevens Creek Blvd, Santa Clara, CA 95051 USA; 4 Key Laboratory of Medical Molecular Virology of Ministries of Education and Health, Basic Medical College, Fudan University, Shanghai, 200032 China; 5 Lindsley F. Kimball Research Institute, New York Blood Center, NY, 10065 USA; 6 Faculty of Information Technology, Macau University of Science and Technology, Avenida Wai Long, Taipa, Macau China; 7 Department of Rheumatology and Immunology, Peking University People’s Hospital, 11 Xizhimen South Street, Beijing, 100044 China; 8 Division of Rheumatology, Jiujiang First People’s Hospital, Taling North Road 48, Jiujiang, 332000 China; 9 Agilent Technologies Hong Kong Ltd., Suite 2603, 26/F, AXA Tower, Landmark East, Kwun Tong, Hong Kong, China; 10 Guangdong Provincial Hospital of Chinese Medicine, Dade Road 111, Guangzhou, 510120 China

## Abstract

N-linked glycans on immunoglobulin G (IgG) have been associated with pathogenesis of diseases and the therapeutic functions of antibody-based drugs; however, low-abundance species are difficult to detect. Here we show a glycomic approach to detect these species on human IgGs using a specialized microfluidic chip. We discover 20 sulfated and 4 acetylated N-glycans on IgGs. Using multiple reaction monitoring method, we precisely quantify these previously undetected low-abundance, trace and even ultra-trace N-glycans. From 277 patients with rheumatoid arthritis (RA) and 141 healthy individuals, we also identify N-glycan biomarkers for the classification of both rheumatoid factor (RF)-positive and negative RA patients, as well as anti-citrullinated protein antibodies (ACPA)-positive and negative RA patients. This approach may identify N-glycosylation-associated biomarkers for other autoimmune and infectious diseases and lead to the exploration of promising glycoforms for antibody therapeutics.

## Abstract (web summary)

Post-translational modifications can affect antibody function in health and disease, but identification of all variants is difficult using existing technologies. Here the authors develop a microfluidic method to identify and quantify low-abundance IgG N-glycans and show some of these IgGs can be used as biomarkers for rheumatoid arthritis.

## Introduction

Glycans can affect the clearance of pathogens and toxins as the specificity of IgG variable regions are coupled to Fc-mediated cellular functions which associate closely with Fc-linked glycans^1, 2^. In particular, IgG glycoforms have been used as molecular signatures for the diagnosis of various diseases, such as rheumatoid arthritis (RA), and the prediction of immune responses^2–8^. Glycosylation also has an important effect on the potency and safety of therapeutic antibodies, owing to effects on activity and possibly also stability, solubility, clearance and immunogenicity of antibody-based drugs, such as intravenous immunoglobulin (IVIG) and monoclonal antibodies (mAb)^9^. Therefore, the identification of functional N-glycans is a promising area of biomedical research^1, 10^.

Glycans are encoded in a complex dynamic network of hundreds of genes that participate in the biosynthetic pathway of protein glycosylation, resulting in the generation of extremely complicated and variable glycosylation profiles, even for single glycoproteins^11, 12^. Such heterogeneity of glycan structures means that individual glycoforms may have very low abundance. According to Go et al.^13^, complete glycosylation analysis is a great challenge due to the complexity of samples, wide dynamic range of glycosylation level and structure heterogeneity of glycans. Notably, many low-abundance and trace N-glycans are biologically important, whereas high-abundance N-glycans generally function as ‘safe switches’^11, 14, 15^. Acidic N-glycans with anionic residues, such as sialic acid, sulfate, and phosphate groups, are examples of such low-abundance but biologically important IgG species^16–18^. For example, IgGs with sialylated N-glycans account for a small portion of pooled IgGs, but have anti-inflammatory activity via binding to dendritic cell-specific intercellular adhesion molecule-3-grabbing nonintegrin (DC-SIGNR), rather than Fcγ receptors^9, 16, 19^ In addition, the ionization efficiency of acidic N-glycans is generally lower than that of neutral N-glycans, resulting in much lower signal intensities^18, 20, 21^. Therefore, a comprehensive glycomic approach to detect low-abundance and difficult-to-detect, but biologically important, species is required.

Although mass spectrometers with enhanced sensitivity exist, the overall increased signal intensity can lead to elevated ion-suppression/interference arising from high-abundance neutral N-glycans/matrix^11^. Chromatographic enrichment is essential for improving the detection sensitivity of low-abundance acidic N-glycans and glycoproteins; however, it has not been done on a micro scale^11^. To microminiaturize and automate the process, we design a microfluidic chip in which a titanium dioxide (TiO_2_) column enriches acidic N-glycans and a porous graphitized carbon (PGC) column chromatographically separates N-glycans. This design enables the comprehensive profiling and accurate quantification of N-glycans, including extremely low-abundance N-glycans, and particularly the acidic species.

## Results

### Design and workflow of TiO_2_-PGC chip

**Fig. 1:**
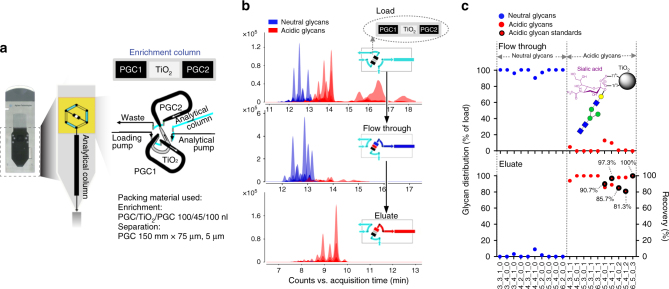
On-chip enrichment of acidic N-glycans. **a** Diagram of the specialized TiO_2_-PGC chip. **b** Extracted compound chromatograms (ECC) of N-glycans detected in the load, flow-through (neutral N-glycans) and eluate fractions (acidic N-glycans) of the TiO_2_ enrichment column as analyzed by liquid chromatography coupled with nanoelectrospray ionization quadrupole time-of-flight mass spectrometry in the positive mode. **c** TiO_2_ enrichment column selectively captures acidic N-glycans. Neutral N-glycans (*blue dot*) do not efficiently bind to TiO_2_ and are recovered in the flow-through fraction, whereas acidic N-glycans are retained on TiO_2_ during the load and flow-through steps and are eluted with the elution buffer. The enrichment recovery rates (*numbers beside the dots*) of five acidic N-glycan standards (*red dots* with *black circles*) are also given

The multilayered polymeric microfluidic chip with TiO_2_-based enrichment architecture was first described in 2008 as a two dimensional HPLC separation device for phosphopeptide analysis^22^. This design enabled the integration of two independent separation functions on a single microfluidic device, where each separation function was on a different layer of the device. To develop a feasible and integrated glycomic approach, we have drawn on the multilayer chip concept and expanded it to the design and fabrication of a novel microfluidic chip, TiO_2_-PGC chip. TiO_2_ has a high affinity for negatively charged molecules^23–26^. Accordingly, the rapid and highly selective on-chip enrichment of acidic N-glycans can be achieved using the high selectivity of TiO_2_ toward negatively charged N-glycans. Thus the TiO_2_-PGC chip is particularly designed for acidic N-glycans enrichment and analysis. It contains a “sandwich-like” enrichment column composed of TiO_2_ and PGC (Fig. 1a). This enrichment column is connected to analysis column with PGC stationary phase, ending in an integrated electro-spray tip. The chip cube allows mounting and positioning of the chip tip.

During analysis, a micro valve is connected directly with the chip surface to create a zero dead volume via high-pressure seal. First, the sample is loaded onto the chip and all N-glycans are retained on the first PGC section. Second, using a linear gradient H_2_O/acetonitrile (ACN) as mobile phase, all N-glycans are eluted off the first PGC enrichment column. Neutral N-glycans pass the TiO_2_ section and the second PGC enrichment column and get transferred onto the analytical column on which they are separated. During this process, acidic N-glycans are retained and enriched on the TiO_2_ column. Third, by a plug of elution buffer (NH_3_), those retained acidic N-glycans are desorbed and retained onto the second PGC enrichment column, and are transferred to the analytical column by subsequent gradient elution.

### Improved detection of acidic N-glycans by on-chip enrichment

The “sandwich” design of the enrichment column on the TiO_2_-PGC chip enables the enrichment of N-glycans on TiO_2_ after a continuous “pre-fractionation” on PGC (PGC1, Fig. 1a), thus allowing for the enrichment of trace species from a complex N-glycan pool. Using a complicated mixture obtained from serum IgGs (Supplementary Fig. 1), we separated low-abundance acidic N-glycans from much more abundant neutral N-glycans with high selectivity (>80%, Fig. 1b, c) on this specialized chip. The coupling of this capability with the relatively high binding capacity of the enrichment column (Supplementary Fig. 2a) facilitated a broad and dynamic range of on-chip enrichment, as indicated by the quantitative enrichment of acidic species from total N-glycans of 0.03–0.5 μg IgG (Supplementary Fig. 2b). These features make this chip feasible for analyzing various biological samples in which the level of N-glycans may vary greatly.

Compared with the offline Strong Anion Exchange (SAX) method, the number of acidic N-glycans enriched using this online chip increased by about 50% (Supplementary Fig. 3a), and an overall improvement in reproducibility was demonstrated (Supplementary Fig. 3b–d). Notably, the relative abundances of N-glycans with varied numbers of sialic acids were conserved after enrichment, indicating that an enrichment bias was not introduced and a “natural and genuine” profile of acidic N-glycans was achieved (Supplementary Fig. 4).

**Fig. 2:**
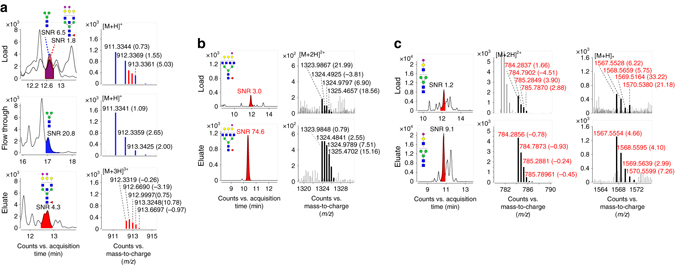
The greatly improved detection of acidic N-glycans after enrichment on the TiO_2_-PGC chip. In **a**, the [M+3H]^3+^ ions of an acidic N-glycan were overlapped with the isotopic ions of co-eluted neutral N-glycans and could not be assigned accurately. Using the TiO_2_-PGC chip, the acidic N-glycans were enriched efficiently and detected with an enhanced S/N and improved mass accuracy because of the removal of interference from neutral N-glycans. In **b**, the signal intensity as well as the S/N of acidic N-glycans were significantly enhanced after on-chip enrichment because of the reduced ion-suppression derived from neutral N-glycans/matrix. In **c**, with the removal of noise derived from neutral N-glycans/matrix, the acidic N-glycan mass accuracy was significantly enhanced, thus providing reliable evidence for the identification of acidic N-glycans. The number in the bracket beside each mass value indicates the mass error (p.p.m.)

Using the TiO_2_-PGC chip, the detection of low-abundance acidic N-glycans was achieved with improved sensitivity because of the decreased ionization suppression derived from otherwise co-eluted neutral N-glycans and/or other matrix components, as demonstrated by the up to 25-fold increase in the signal-to-noise ratio (S/N) of acidic N-glycans after enrichment (Fig. 2, chromatograms). In addition, the online enrichment enabled enhanced mass accuracy and isotope distribution of the intact N-glycan’s ion because of the removal of neutral N-glycans and/or other molecules with similar molecular weights (Fig. 2, MS spectrum). Of note, the enrichment procedure facilitated reliable tandem Mass Spectrometry (MS/MS) measurements by removing isomeric and/or isobaric ions that can pass through the quadrupole to undergo fragmentation and compromise the fragmentation information.

### Development of dual-mobile phase approach

In a subsequent step of method optimization, we attempted to use specific mobile phase to separately improve the detection of neutral and acidic N-glycans. To this end, we developed a “dual mobile phase” approach in line with the special work mode of TiO_2_-PGC chip. We examined different mobile phases for the analysis of neutral and acidic N-glycans individually, and then used optimal mobile phase for respective class of N-glycans. In the finalized dual-mobile phase approach, 1% formic acid (FA) in water (A1) and acetonitrile (ACN) (B1) were used as mobile phase of the first run (for the analysis of neutral and high mannose N-glycans), while 0.5% FA in water (adjusted to pH 3 by ammonia solution) (A2) and 1% FA in ACN (B2) were employed as the mobile phase of the second run (for the analysis of acidic N-glycans). The dual-mobile phase approach improved the detection sensitivity of three types of N-glycans by approximately 5-fold as compared to routinely used single mobile phase (Supplementary Fig. 5). This benefit, together with the improvements achieved by online enrichment, provided a firm basis for the detection of low-abundance acidic N-glycans, which cannot be achieved by simply enhancing the overall signal intensity. As such, the dual-mobile phase approach was applied to both HPLC chip-QTOF MS system for N-glycan profiling and HPLC chip-QQQ-MS system for quantitative N-glycan analysis.

### Comprehensive N-glycan profile of human serum IgGs

**Fig. 3:**
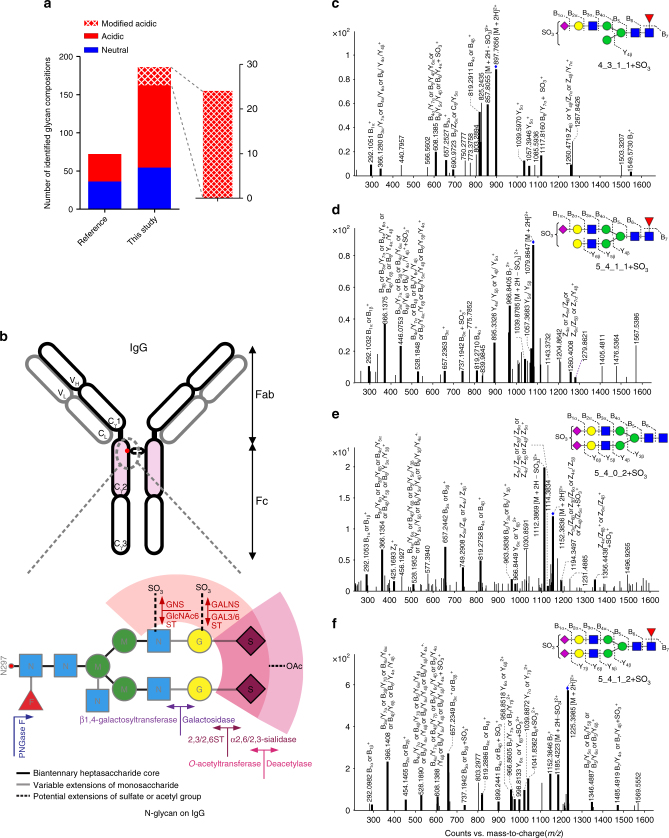
Comprehensive profiling of N-glycans on serum IgGs. **a** The number of N-glycans characterized on serum IgGs using the TiO_2_-PGC chip coupled with Q-TOF MS. **b** Structural maps of N-glycans on IgG. **c**–**f** MS/MS of representative sulfated N-glycans

The specialized TiO_2_-PGC chip, combined with dual-mobile phase method, facilitated an integrated glycomic approach that enabled the most comprehensive profiling of N-glycans on IgGs reported thus far. Using ~ 2.5 μg serum polyclonal IgGs, a total of 444 N-glycans generated from 185 distinct compositions were identified in the current study, including 54 and 131 compositions of neutral and acidic N-glycans, representing 56% coverage of the recently developed theoretical N-glycan library for serum (331 compositions)^27^. Notably, our identifications almost doubled the number of known neutral N-glycans of IgG (36 compositions) and almost quadrupled the number of acidic N-glycans previously identified on IgG (36 compositions; Fig. 3a)^28^. The newly identified N-glycans not only demonstrated a sharp increase in the detection capability of acidic N-glycans via the on-chip method but also suggested a remarkable and previously unknown structural diversity of acidic N-glycans on IgG.

**Fig. 4:**
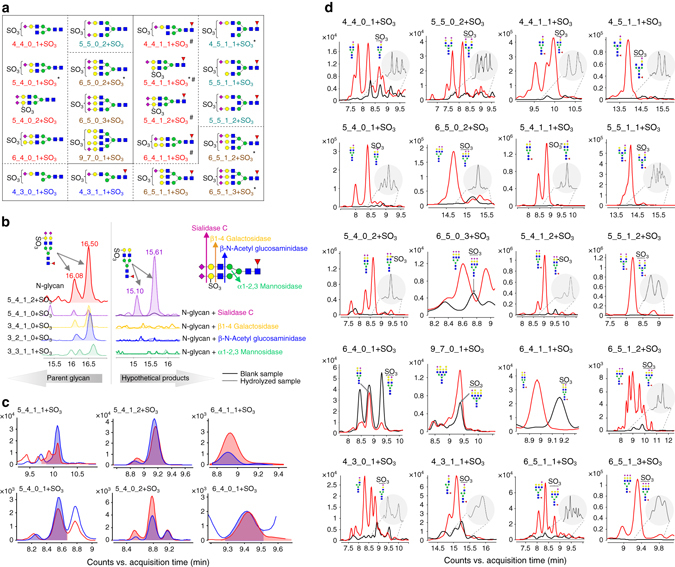
Structures of sulfated N-glycans identified on human serum IgGs. **a** Structures of identified sulfated N-glycans in human serum IgG. (*represents sulfated N-glycans that have been reported from a human source; ^#^represents sulfated N-glycans that have been reported on porcine thyroglobulin; another 11 sulfated N-glycans have not been previously reported). **b** Four exoglycosidases, Sialidase C, *β*1–4 galactosidase, *β*-N-acetyl glucosaminidase, and *α*1–2, 3 mannosidase, were employed to hydrolyze the sulfated N-glycans to determine site of the sulfate group. **c** The retention times and peak patterns of sulfated N-glycans in human IgGs (*red*) were compared with those in porcine thyroglobulin (*blue*), which has been well characterized. **d** The retention time of each sulfated N-glycan (*black*) is 0.5 min later than its corresponding parent N-glycans (*red*), and the signal intensity is 3-fold to 200-fold lower than that of the non-sulfated N-glycans

The comprehensive profiling of acidic N-glycans resulted in an enlarged bioinformatics framework of N-glycans that can be attributed to the variation in saccharide compositions as indicated by multiple fucosylated (2–3) ternary, quaternary or pentanary N-glycan structures, and the modification of the N-glycan structures as represented by *O*-acetylation and sulfation which has never been observed on human serum glycoproteins (Fig. 3b, Supplementary Data 1, 2). More importantly, many sulfated N-glycans (36 structures arising from 20 distinct compositions) were discovered on IgGs for the first time, including 9 compositions that were previously found in recombinant human tissue plasminogen activator^29^, human urine^30^ and on porcine thyroglobulin^31, 32^, respectively, and 11 compositions that had not been previously reported (Fig. 4a).

The 20 sulfated N-glycan compositions were primarily assigned based on their high-resolution MS data (Supplementary Fig. 6, Supplementary Data 1). The presence of sulfate group was confirmed by the observation of diagnostic fragment ion originating from in-source neutral loss [M+2H-SO_3_]^2+^ or [M+3H-SO_3_]^3+^ (Supplementary Fig. 6c). The structures of the “net” N-glycan part were verified by high-resolution MS/MS data (Supplementary Data 1) for 19 compositions.

Of note, fragment ions with sulfate group were observed for 8 out of the 20 compositions in high-resolution MS/MS spectra, further demonstrating sulfation of these N-glycans. In addition, these fragments provided key information for the location of sulfate group. Take Hex_5_HexNAc_4_dHex_1_NeuAc_2_ + SO_3_ (5_4_1_2 + SO_3_) as an example, fragment ions corresponding to “[B7/Y4 + SO_3_]^+^” (*m*/*z* 1485.4919), “[Y6 + SO_3_]^2+^” (*m*/*z* 998.8133), “[B4 + SO_3_]^+^” (*m*/*z* 899.2441), “[B3 + SO_3_]^+^” (*m/z* 737.1942) and “[B4/Y7 + SO_3_]^+^” (*m/z* 608.1388) were clearly observed in the MS/MS spectrum of the [M + 2 H]^2+^ at *m/z* 1225.3985, suggesting that the sulfate group was located on galactose or N-acetyl glucosamine (Fig. 3c–f). This assignment was supported by exoglycosidase experiment which suggested the attachment of the sulfate group on galactose of *α*1–3 branch (Fig. 4b).

In addition, a total of six sulfated N-glycans have been reported in porcine thyroglobulin (pTG), with NMR confirmed structures. We employed N-glycan mixture released from pTG as “authentic” sample and verified the identification of these sulfated N-glycans on IgGs (Fig. 4c). In addition, all of the sulfated N-glycans exhibited consistent retention time differences (typically + 0.5 min) relative to their “parent” species, and their signal intensities were 3-fold to 200-fold lower than those of their non-sulfated counterparts (Fig. 4d). The specific retention behavior can be employed as a supporting evidence for the identification of sulfated N-glycans. Sulfation constitutes a novel structural variation and represents another type of N-glycan microheterogeneity on IgG.

### Quantitative glycomic approach by MRM

**Fig. 5:**
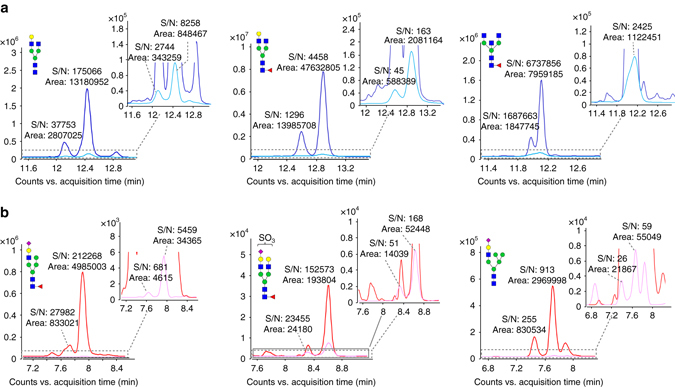
Improved detection of N-glycans using the MRM method. N-glycans were detected using Q-TOF MS (*pale blue* or *pale red*) and QQQ MS (MRM mode, *blue* or *red*). The improved detection of representative neutral N-glycans Hex_4_HexNAc_4_, Hex_4_HexNAc_4_dHex_1_ and Hex_3_HexNAc_5_dHex_1_ are shown in **a**, and the improved detection of representative sialylated N-glycans Hex_5_HexNAc_3_dHex_1_NeuAc_1_, Hex_5_HexNAc_4_dHex_1_NeuAc_1_ + SO_3_ and Hex_6_HexNAc_3_NeuAc_1_ are shown in **b**. The signal intensities of N-glycans obtained using the MRM method were increased by up to 180-fold compared with those obtained with Q-TOF MS. Notably, the S/N of the acidic N-glycans obtained using MRM increased by ~10-fold to 1000-fold compared with that obtained with Q-TOF MS

The multiple reaction monitoring (MRM) method was then used to quantify the N-glycans of IgGs (Supplementary Data 3), further improving the signal intensity of those low-abundance acidic N-glycans by ~1000-fold compared with that of TOF-MS (Fig. 5, Supplementary Table 1). In particular, the use of TiO_2_ enrichment enabled the highly sensitive detection of acidic N-glycans mixed with complex neutral N-glycans (Supplementary Fig. 7). This quantitative method demonstrated much wider linearity (typically 250-fold to 2000-fold) compared with TOF-MS (generally 30-fold to 70-fold) (Supplementary Table 2). With the MRM method, acidic N-glycan signal intensities can be pointedly enhanced by increasing the dwelling time, thus significantly reducing ionization bias (Supplementary Fig. 8). This MS/MS-based method was further validated for its recovery rate and selectivity (Supplementary Table 3).

**Fig. 6:**
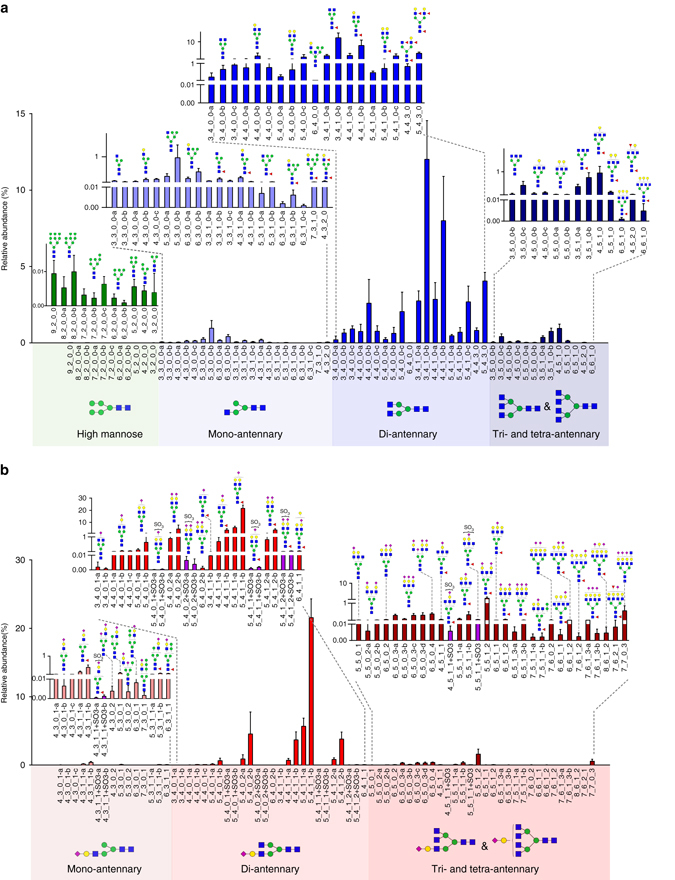
Glycomic analysis of human serum IgGs using MRM method. It revealed a highly dynamic range of up to five orders of magnitude in the relative abundances of both neutral **a** and acidic **b** N-glycans, data are shown as mean ± s.d

The glycome of human serum IgGs were quantitatively profiled by using the established MRM method. The result showed that both neutral and acidic N-glycans presented in IgG of human serum were predominantly in complex type, only less than 0.1% N-glycans were in high mannose type and about 1.8% neutral N-glycans and 0.2% acidic N-glycans were in hybrid type. Among the complex N-glycans, bi-antennary N-glycans occupied the highest portion (90% of overall N-glycans), and most of them were fucosylated (more than 75%) and sialylated (more than 40%), trace acidic N-glycans were modified by a sulfate group (about 1%; Fig. 6, Supplementary Data 4). The quantitative glycomic profiling revealed remarkable depth in the concentration of individual N-glycans on human serum IgGs.

### N-glycan profile of serum IgGs from RA patients

To explore the potential biological role of acidic N-glycans, we extended our analysis to serum samples from RA patients. The entire N-glycomes of the serum IgGs of 90 RA patients and 57 healthy individuals were primarily analyzed, and the relative abundances of individual N-glycans were employed as variations to predict the grouping of individuals. As shown in Supplementary Fig. 9, a general trend of “the more minor, the more widely changed” was observed for both neutral and acidic N-glycans.

For the identification of potential N-glycan biomarkers, we selected informative N-glycan biomarkers (attributes) by using a popular machine learning tool, Weka. The process was performed by employing attribute evaluator by which attribute subsets are assessed and search method with which the space of possible subsets are searched. We used correlation-based feature subset selection method (CfsSubsetEval) to select value subsets that correlate highly with the class value and lowly with each other. And we used the best-first search strategy to navigate attribute subsets in search space. We then fed the classification ready pre-processed data from the entire N-glycome of the serum IgGs of 90 RA patients and 57 healthy samples (training data) into Weka. As the results, 21 N-glycan biomarkers were selected. Subsequently, these 21 biomarkers were used to construct a diagnostic model by using Support Vector Machine (SVM) method individually.

**Fig. 7:**
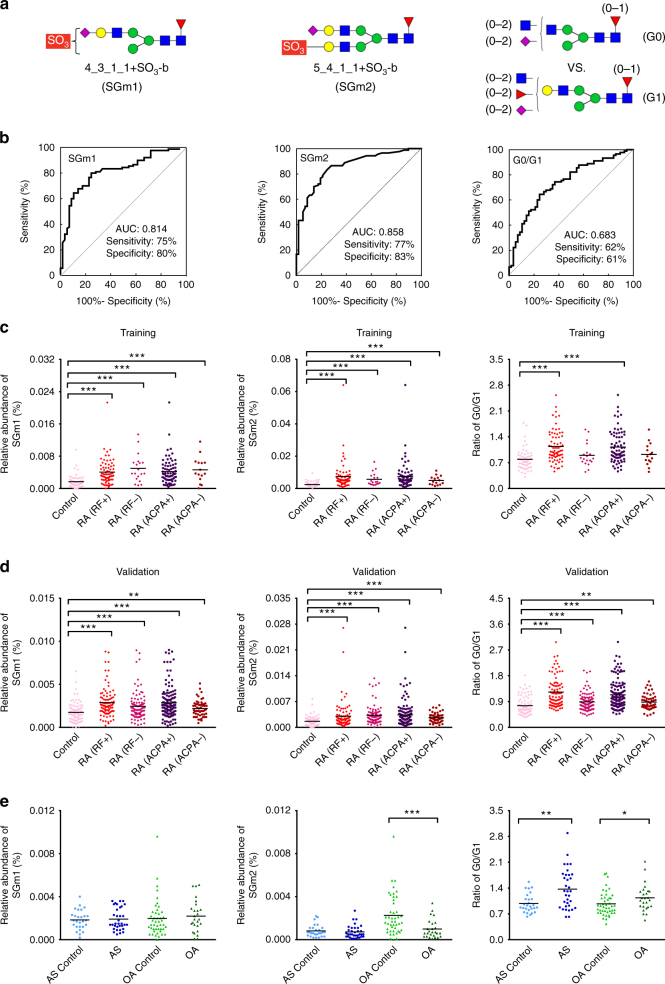
Performance and relative abundances of the potential N-glycan biomarkers for RA. **a** Symbols depicting N-glycan biomarkers identified in current study (SGm1 and SGm2) and that reported previously (G0/G1). **b** The ROC curve of the biomarkers for the classification of RA (*n* = 90) and healthy individuals (*n* = 57). The results are plotted for the training set. **c**, **d** The scatter plot of the relative abundance of the N-glycan biomarker in control individuals (training set: *n* = 57, validation set: *n* = 84), RF-positive [RA (RF+)] (training set: *n* = 71, validation set: *n* = 90) and RF-negative [RA (RF−)] RA patients (training set: *n* = 19, validation set: *n* = 97), as well as ACPA-positive [RA (ACPA+)] (training set: *n* = 75, validation set: *n* = 132) and ACPA-negative [RA (ACPA−)] RA patients (training set: *n* = 15, validation set: *n* = 55), in training set and validation set. **e** The scatter plot of the relative abundance of the N-glycan biomarkers in AS patients (*n* = 34), OA patients (*n* = 26) and in controls of each (AS: *n* = 27, OA: *n* = 45). Horizontal lines indicate the median. Statistical analysis was performed by non-parametric Mann–Whitney test, two-sided (*P < 0.05, **P < 0.01, ***P < 0.001)

The prediction models were measured for sensitivity and specificity as described in the method. For the individual 21 N-glycan biomarkers, the sensitivity values ranged from 58 to 93%, the specificity values varied from 32 to 83%, while the area under the curve (AUC) values ranged from 0.601 to 0.858 (Table 1). Receiver operating characteristic (ROC) curves were further constructed for the identified N-glycan biomarkers, which generated an overall sensitivity of 82 % and a specificity of 78% combined with an AUC of 0.869. Notably, four sulfated N-glycans were identified as biomarkers that distinguish RA patients from the controls (Supplementary Fig. 9c, f). Among them, two sulfated N-glycans, 4_3_1_1 + SO_3_-b and 5_4_1_1 + SO_3_-b, yielded high individual AUCs of 0.814 and 0.858 respectively, as well as relatively high individual sensitivity (75% and 77%, respectively) and specificity (80% and 83% respectively) (Table 1 and Fig. 7a, b), indicating their particular potential value in the classification of RA. Thus, these two sulfated glycans were assigned as high-potential biomarkers for RA and termed as SGm1 and SGm2.

**Table 1:** Capacities of 21 N-glycan biomarkers for the classification of RA patients and healthy individuals

N-Glycan biomarkers	Training set (RA patient (*n* = 90) and healthy individuals (*n* = 57))			Validation set (RA patient (*n* = 187) and healthy individuals (*n* = 84))		
	AUC	Sensitivity (%)	Specificity (%)	AUC	Sensitivity (%)	Specificity (%)
*Neutral N-glycans*						
5_3_1_0-b	0.853	75	82	0.793	93	69
6_3_1_0-b	0.814	77	76	0.768	80	75
6_3_1_0-a	0.758	93	54	0.701	94	53
3_5_0_0-c	0.746	72	69	0.701	75	66
5_5_0_0-b	0.737	58	82	0.734	62	82
5_3_1_0-c	0.736	74	62	0.708	81	64
3_3_0_0-a	0.717	89	57	0.723	90	59
4_3_1_0-b	0.696	60	74	0.723	66	77
4_2_0_0	0.747	81	60	0.738	87	64
*Acidic N-glycans*						
8_6_1_2	0.803	82	71	0.779	84	73
4_3_1_1-a	0.791	81	62	0.786	60	92
4_3_0_1-c	0.758	86	56	0.705	87	59
5_5_0_2-b	0.740	70	69	0.705	73	69
6_5_0_2	0.724	84	53	0.705	75	68
7_5_1_1-b	0.690	79	60	0.705	82	62
5_4_0_1-a	0.657	93	32	0.539	93	25
4_4_1_1-a	0.601	68	56	0.661	53	76
*Sulfated N-glycans*						
5_4_1_1 + SO_3_-b (SGm2)	0.858	77	83	0.819	79	84
4_3_1_1 + SO_3_-b (SGm1)	0.814	75	80	0.793	80	79
5_4_1_2 + SO_3_-b	0.721	67	71	0.697	69	70
5_4_0_1 + SO_3_-a	0.683	81	52	0.664	85	53
All	0.869	82	78	0.804	82	79
SGm1 and SGm2	0.879	84	86	0.849	85	85

To maximize the ability of the N-glycan biomarkers for distinguishing RA patients from healthy individuals, we examined the classification abilities of pair biomarker combinations. Among all plausible combinations (Supplementary Data 5), combination of the two sulfated N-glycan biomarkers (SGm1 and SGm2) generated an optimal capacity for the classification of RA in training set (with AUC of 0.879, sensitivity of 84% and specificity of 86%).

We further examined the accuracy of individual biomarkers for the classification of RF-negative patients in training set (*n* = 19). All N-glycan biomarkers except for 4_2_0_0 were shown to have relatively high prediction capacity (accuracy ≥ 84%) with an overall accuracy of 89%. As ACPA is also ubiquitous in clinical care, we also examined the performance of the biomarkers for the classification of ACPA-negative patients (*n* = 15), as well as RF&ACPA-negative patients (*n* = 8). As shown in Table 2 and Fig. 7, the identified 21 biomarkers showed generally high prediction capacity for ACPA-negative and RF&ACPA-negative patients, with an overall prediction accuracy of 87 and 100% in training set respectively. The combination of SGm1 and SGm2 yielded prediction accuracy of 95, 93 and 95% for RF-negative, ACPA-negative and RF&ACPA-negative patients.

**Table 2:** Prediction accuracy (%)^d^ of 21 N-glycan biomarkers for RF-negative and/or ACPA-negative patients

N-Glycan biomarkers	Training set			Validation set		
	R(−)^a^ patients (*n* = 19)	A(−)^b^ patients (*n* = 15)	R&A(−)^c^ patients (*n* = 8)	R(−)^a^ patients (*n* = 97)	A(−)^b^ patients (*n* = 55)	R&A(−)^c^ patients (*n* = 45)
*Neutral N-glycans*						
5_3_1_0-b	95	93	100	88	91	96
6_3_1_0-b	100	100	100	77	82	80
6_3_1_0-a	95	93	100	72	69	76
3_5_0_0-c	100	100	100	79	82	82
5_5_0_0-b	89	93	100	66	60	60
5_3_1_0-c	95	93	100	77	87	91
3_3_0_0-a	95	93	100	71	58	64
4_3_1_0-b	95	93	100	69	58	64
4_2_0_0	63	53	50	82	95	93
*Acidic N-glycans*						
8_6_1_2	89	87	75	80	75	78
4_3_1_1-a	95	93	88	75	75	76
4_3_0_1-c	89	93	88	78	82	84
5_5_0_2-b	100	100	100	71	65	76
6_5_0_2	89	93	88	72	73	76
7_5_1_1-b	89	87	75	77	78	82
5_4_0_1-a	100	100	100	59	51	60
4_4_1_1-a	89	87	88	59	51	60
*Sulfated N-glycans*						
5_4_1_1 + SO_3_-b (SGm2)	95	93	88	84	82	82
4_3_1_1 + SO_3_-b (SGm1)	100	100	100	76	78	73
5_4_1_2 + SO_3_-b	95	93	100	64	51	60
5_4_0_1 + SO_3_-a	84	80	88	70	82	82
All	89	87	100	81	80	80
SGm1 and SGm2	95	93	95	87	85	84

^a^R(−) represented RF-negative RA patients

^b^A(−) represented ACPA-negative RA patients

^c^R&A(−) represented RF&ACPA-negative RA patients

^d^The prediction accuracy refers to the ratio of the number of samples correctly predicted as RA to the total number of samples actually diagnosed as RA in corresponding subsets using the regular diagnostic methods

The sensitivity and specificity for the biomarkers are measured by identifying the cutoff point of the predicted probability that yielded the highest sum of sensitivity and specificity. For verification^33, 34^, all the 21 biomarkers and its corresponding cut off points were applied to a validation set consisted of 187 RA patients and 84 healthy samples. This independent, blinded analysis confirmed the performance of the biomarkers established in training set, as demonstrated by an overall AUC of 0.804, sensitivity of 82% and specificity of 79%. Notably, the prediction capacity of these biomarkers for RF- and/or ACPA-negative RA patients were verified in a much bigger subset of the validation cohort (*n* = 97 for RF-negative patients, *n* = 55 for ACPA-negative patients, and *n* = 45 for RF&ACPA-negative patients), with an overall prediction accuracy of 81%, 80% and 80%, respectively. Furthermore, performance of SGm1, SGm2 and their combination were validated by their comparable AUC, sensitivity, specificity, and prediction accuracy for RF and/or ACPA-negative patients in the validation set as that in training set (Tables 1, 2).

In addition, although some differences of clinical features between the training set and the validation set were observed, the values in both data sets felled into the range of clinical settings, thus reflected the clinical heterogeneity of RA patients. Validation on such heterogeneous data sets just allowed for the evaluation of the generalizability of the established model to wider RA populations.

As RF-independent biomarkers, SGm1 and SGm2 may be used for the diagnosis of autoantibody-negative RA patients. To this end, we further examined the specificity of SGm1 and SGm2 for RF/ACPA-negative RA patients by analyzing patients of another RF/ACPA-negative inflammatory disorder, ankylosing spondylitis (AS, *n* = 34), as well as a degenerative joint disease osteoarthritis (OA, *n* = 26). As shown in Fig. 7c–e, SGm1, which showed significantly enhanced relative abundance in both RF-positive and –negative RA patients, displayed almost unchanged levels in AS and OA patients as compared to their age and gender-matched healthy controls. Of interest, SGm2 exhibited disease-associated changes in RA, OA and AS (Fig. 7c–e). It showed significantly elevated levels in RA patients, obvious reduced levels in OA patients, and no significant changes in AS patients, as compared to age and gender-matched healthy controls of each. These findings confirmed the specificity of SGm1 and SGm2 upregulation for RA patients, regardless they are RF/ACPA-positive or negative.

Compared with previously reported serum N-glycan biomarkers, such as the ratio of G_0_/G_1_ (Fig. 7c–e)^3–5, 10, 35^, the newly identified N-glycan biomarkers, especially SGm1 and SGm2, exhibited higher potential for the classification of RF/ACPA-negative RA patients, (Table 2). Of note, comparing to other disease controls, no specific change was observed for G_0_/G_1,_ as indicated by the observation of significant increased levels in both AS and OA as compared to respective controls, showing that this biomarker was not specific for RA patients.

## Discussion

Any changes in the structures or levels of even trace N-glycans can result in significant physiological/pathological events. By sharply increasing the glycome coverage and depth, our chip-based approach provides an early glimpse into the remarkable structural complexity of N-glycans resulting from microheterogeneity expressions, such as sulfation, phosphorylation and acetylation. Moreover, as all N-glycans share a common core sugar sequence, the TiO_2_-PGC chip-based glycomic approach is applicable for profiling N-glycans released from any single glycoprotein or total glycoproteins. N-glycosylation occurs on numerous secreted and membrane-bound glycoproteins^12^, and N-glycan components are often the crucial functional determinants of biological events. Therefore, our glycomic approach will rapidly position itself as one of the most important tools for addressing certain key biological and pathological questions. Furthermore, because of the conserved biosynthesis of N-glycans across metazoa, plants, yeast and even bacteria^12^, this on-chip glycomic approach can be further extrapolated to the quality control of antibody-based drugs^1^ and design of vaccines because antigen glycosylation, including N-glycosylation, has been increasingly appreciated as essential in adaptive immune activation^36^.

Our study represents the first glycomic method that enables the detection of low-abundance and even trace novel sulfated N-glycans on IgGs. Because of the conserved biosynthesis of N-glycans, the novel sulfated N-glycans are most likely present on other glycoproteins. It was believed that sulfation of N-glycans can significantly alter biological recognition and/or facilitate rapid clearance of the protein from the body^37–40^. Especially, N-glycans with sulfo-modifications constitute important recognition codes in cell adhesion, e.g., a glucosamine containing sulfate group in position O-6 and an N-acetyl group was the preferred epitope for the immune recognition^31, 41–43^. The effect of sulfated N-glycans on IgG is yet to be known. However, as terminal sugar of N-glycan may affect the function of IgG dramatically via altering the structure of Cγ2 domain, we speculated that sulfated N-glycans on IgG may induce significant structural alterations in Cγ2 domain, and subsequently change the ligand specificity and biological functions of IgG, which need to be studied in the future.

Next to the early-appreciated correlation between hypogalactosylation of serum total IgGs and the severity of RA, more attention have been made on the analysis of specific glycan profile of ACPA antibodies that is distinct from the profile of total serum IgGs^44, 45^. Furthermore, increasing experimental evidence points to a critical role of autoantibody-associated glycosylation of IgGs in RA. For instance, extensive glycosylation of ACPA-IgG variable domains were found to modulate binding to citrullinated antigens in RA^3^, while ACPA antibody itself were demonstrated to acquire a pro-inflammatory Fc glycosylation phenotype prior to the onset of RA^46^. In addition, agalactosyl glycoforms of IgG autoantibodies were suggested to be pathogenic for RA^35, 47^. Despite these findings, specific glycosylation-based biomarkers with diagnostic capabilities for autoantibody-negative RA are still absent^48^. In this context, the trace N-glycan biomarkers identified in our study, especially SGm1 and SGm2, could have important clinical implications for the diagnosis of RA as they are RF/ACPA-independent and RA-specific. In addition, as N-glycan biomarkers of total IgG rather than autoantibody-specific IgG, the on-chip method readily lends itself to clinical applications. Even though, for the discrimination of RA from other inflammatory arthritis which is a crucial clinical question^49–51^, further studies need to be performed to address the specificity of SGm1 and SGm2.

In summary, we have developed an innovative platform based on TiO_2_-PGC chip for in-depth glycomic research of human serum IgGs. This innovative approach facilitated the identification of a high number of glycans on IgGs and the discovery of a number of sulfated glycans on IgGs for the first time. We have also demonstrated the significance of this cutting-edge glycomic platform in discovering potential biomarkers of RA disease. Two sulfated glycans have been proved to exhibit notably high capacity for the classification of both rheumatoid factor (RF)-positive and negative RA patients, as well as anti-citrullinated protein antibodies (ACPA)-positive and negative RA patients, showing notable and specific value of N-glycan as biomarkers for RA. Application of this novel approach in a wider range of glycomic research may reveal potential N-glycosylation-associated biomarkers for other autoimmune and infectious diseases. Further studies on the biosynthesis and biological functions of novel sulfated glycans identified in this study may contribute potentially to the pathological study of autoimmune diseases.

## Methods

### Patients and collection of serum samples

A total of 141 healthy individuals and 277 RA patient samples from three independent set were analyzed in this study, comprising a training set and validation set. The clinical features of RA patients and healthy individuals were shown in Table 3. The training set consisted of 57 healthy individuals and 90 RA patient samples (including a small subset of RF-negative patients (*n* = 19) and subset of ACPA-negative patients (*n* = 15)). The samples were collected from the Division of Rheumatology of Jiujiang No. 1 People’s Hospital (Jiujiang, China). The validation set consisted of 84 healthy individuals and 187 RA patient samples (including a bigger subset of RF-negative patients (*n* = 97) and subset of ACPA-negative patients (*n* = 55)). Samples were obtained from Jiujiang and the Department of Rheumatology and Immunology of Peking University People’s Hospital (Beijing, China).

**Table 3:** Clinical features of RA patients and healthy individuals

Clinical features	Training set			Validation set		
	RA patients (*n* = 90)	Healthy individuals (*n* = 57)	*P*-value^a^	RA patients (*n* = 187)	Healthy individuals (*n* = 84)	*P*-value^a^
Age, years	57.2 ± 13.6	56.7 ± 14.2	0.85	55.8 ± 12.7	52.9 ± 10.8	0.07
Gender (female), %	84.4	71.9	0.37	75.9	67.9	0.18
Disease duration, years	7.4 ± 7.1	ND		7.3 ± 6.8	ND	
*Serology parameters:*						
RF positive, %	78.9	ND		48.1^b^	ND	
RF, IU ml^−1^	410.3 ± 695.5	ND		376.6 ± 672.8	ND	
ACPA positive, %	83.3	ND		70.6	ND	
ACPA, units per ml	717.6 ± 698.5	ND		840.5 ± 1115.4	ND	
ESR, mm h^−1^	48.6 ± 31.8	ND		42.3 ± 29.1	ND	
CRP, mg l^−1^	2.5 ± 3.2	ND		10.9 ± 21.1	ND	
*Medication used:*						
Leflunomide, %	72.2	ND		58.8	ND	
Methotrexate, %	51.1	ND		47.1	ND	
Hydroxychloroquine, %	24.4	ND		21.9	ND	

*ACPA* anti-citrullinated protein antibodies, *CRP* C-reactive protein, *ESR* erythrocyte sedimentation rate, *ND* not determined, *RF* rheumatoid factor

Data are shown as mean ± s.d.

^a^ *P*-values were calculated by the non-parametric Mann–Whitney test for age and by the Fisher’s exact test for gender, two-sided

^b^RF-negative patients were collected on purpose to confirm the prediction capacity of the biomarkers for RF-negative samples, hence the RF-positive percentage in validation set was much lower than that in training set

All RA patients, including RF-negative and ACPA-negative RA patients, fulfilled the 2010 ACR/EULAR Classification Criteria for Rheumatoid Arthritis. Firstly, all of the patients must meet two requirements: (1) patient with at least 1 joint with definite clinical synovitis (swelling) and (2) the synovitis is not better explained by “another” disease. With the fulfillment of the two requirements, patients with a total score of 6 or greater from the individual scores in 4 domains were classified as definite RA, whereas patients with erosions typical for RA were classified as RA even without application or fulfillment of the scoring system of 2010 ACR/EULAR Classification Criteria for RA^52^. Samples from patients of other RF-negative chronic inflammatory disorders, ankylosing spondylitis (AS, *n* = 34) and osteoarthritis (OA, *n* = 26) and controls of each (*n = *27 for AS; *n = *45 for OA) were collected from the Division of Rheumatology of Jiujiang No. 1 People’s Hospital and Guangdong Provincial Hospital of Chinese Medicine (Guangzhou, China). AS patients were classified according to the modified New York Criteria^53^ and European Spondyloarthropathy Study Group Criteria^54^. All knee OA patients fulfilled the American College of Rheumatology (ACR) classification criteria^55^. The clinical features of AS and OA patients were shown in Supplementary Table 4 and 5.

Serum samples were retrieved from hospital following the same protocol. All sera were stored at −80 °C prior to use. The study was approved by the Ethic Committees of the relevant hospitals and informed consent was obtained from all patients.

### Capture of IgGs from serum samples

IgGs were isolated using Protein A. Briefly, rProtein A Sepharose 4 Fast Flow beads were applied to a 96-well filter plate at 50 µl per well. After washing twice with five volumes of binding buffer (20 mM sodium phosphate, pH 7.0), 250 µl binding buffer and 10 µl serum were successively applied to each well. The plate was sealed and incubated on a shaker at room temperature for 15 min. The filtrate was collected in a V-bottom collection plate by centrifugation (1000 r.p.m., 5 min). The retained beads were washed twice with 250 µl binding buffer. IgGs were then eluted twice with 200 µl elution buffer (0.1 M glycine buffer, pH 2.7) into a new V-bottom collection plate. Next, 30 µl neutralizing buffer (1 M Tris-HCl, pH 9.0) was added for neutralization. The obtained IgG samples were then transferred to 30 K centrifuge filter units for buffer exchange, and the resulting water solutions were concentrated to a final volume of 30 µl. The amount of captured IgGs in each sample was quantitated using a Bio-Rad protein assay. The purity of the captured IgGs was examined using SDS–PAGE and HPLC.

### Release of N-glycans

N-glycans were cleaved by using PNGase F. 50 µg IgGs from each patient sample was diluted with 100 mM ammonium bicarbonate buffer (pH 7.4) to a final concentration of 1 µg µl^−1^. Then, 0.5 µl PNGase F was added, followed by a 16 h incubation at 37 °C. The cleaved N-glycans were directly loaded onto the preconditioned C_18_ cartridge and washed with 1.0 ml distilled water to remove the de-glycosylated protein. The flow-through and water eluates were combined and dried by speed vacuum. The dried residues were reconstituted in 100 µl distilled water and stored at −80 °C before analysis.

### Design and fabrication of TiO_2_-PGC Chip

A customized TiO_2_-PGC chip composed of a sandwich-like enrichment column, which included a TiO_2_ column between two PGC trapping columns, was fabricated. The customized TiO_2_-PGC chip consists of a 75 µm × 150 mm PGC analytical column (PGC, 5 µm) and a three-sectioned enrichment column, including a 100 nl PGC trapping column (PGC 5 µm), a 45 nl TiO_2_ column, and a second 100 nl PGC trapping column (Agilent, Waldbronn, Germany). The sandwich design of the enrichment column allowed for the enrichment of acidic N-glycans on TiO_2_, whereas neutral N-glycans were eluted and analyzed in the first step. The acidic N-glycans were subsequently eluted by injecting an elution buffer (0.5% ammonia solution in water) in the second step. The acidic N-glycan binding capacity of the TiO_2_-PGC chip was evaluated using a breakthrough experiment. The online enrichment performance of the TiO_2_-PGC chip for acidic N-glycans was compared with the offline SAX method.

### HPLC chip/MS

An Agilent 1260 Infinity HPLC Chip LC system was coupled to an Agilent 6550 iFunnel Quadrupole Time-of-Flight (Q-TOF) mass spectrometer (MS) for N-glycan profiling or coupled to an Agilent 6490 iFunnel Triple Quadrupole (QQQ) MS for N-glycan quantification. The Agilent 1260 Infinity HPLC Chip system was equipped with a HiP micro ALS sampler with a 40 µl sample loop, a nanoflow pump, a capillary pump, a HPLC Chip Cube Interface, a thermostat and a µ-degasser. A 25 µm ID PEEK capillary was used for sample transfer to prevent the dissolution of fused silica by the high-pH elution buffer.

### On-chip enrichment of acidic N-glycans

The TiO_2_-PGC chip was operated in the forward flush mode. First, 2 µl of the sample were injected and transferred onto the enrichment column using 0.6% acetic acid, 2% FA and 2% ACN in water at a flow rate of 3 µl min^−1^. The chip valve was switched 2 min after injection to place the enrichment column in-line with the analytical column. The mobile phase used in the nanoflow pump was optimized for neutral N-glycans and consisted of 1% FA in water (A) and ACN (B). The gradient was performed at a flow rate of 0.5 µl min^−1^ as follows: 5% B for 6 min, 5–60% B over 10 min, and 80% B for 3 min. The acidic N-glycans were subsequently eluted by injecting 5 µl elution buffer (0.5% ammonia solution in water). The analysis of the enriched acidic N-glycans was performed by switching the enrichment column in-line with the analytical column 1 min after injection. The mobile phase optimized for the acidic N-glycans was used. Mobile phase A was 0.5% FA in water adjusted to pH 3 with the ammonia solution, and mobile phase B was 1% FA in ACN. The flow rate was 0.5 µl min^−1^, and the gradient was as follows: 5% B for 1 min, 5–60% B over 10 min, and 80% B for 3 min. An equilibrium time of 18 min was set before each injection.

### N-glycan profiling by HPLC chip-Q-TOF MS

The structures of the N-glycans were characterized based on high-resolution MS and MS/MS data obtained on Q-TOF MS in the positive mode. The dry-gas (N_2_) temperature and flow rate were 225 °C and 11 l min^−1^, respectively. The MS spectra were acquired in the positive mode, and the mass range was 500–3000 *m/z* with an acquisition time of 1 spectrum per sec. Mass correction was enabled using reference masses of 922.0098 *m/z* and 1221.9906 *m/z*. The mass range of the MS/MS experiments was 100 to 3000 *m/z*. The spectra were acquired in the targeted MS/MS mode with an MS acquisition rate of 2 spectra per sec and an MS/MS acquisition rate of 3 spectra per sec. The collision energy (CE) was set to 10–40 eV.

### Quantitative profiling of N-glycans on HPLC Chip-QQQ-MS

The released N-glycans were quantitatively profiled on Chip-QQQ-MS using MRM method in positive mode. The dry-gas (N_2_) temperature and flow rate were 225 °C and 11 l min^−1^, respectively. The RF voltage amplitude of the high-pressure and low-pressure ion funnels were 150 and 200 V, respectively. The dwell time was set as 10–50 ms. All of the raw data were processed using Agilent MassHunter Qualitative Analysis B.06.00 and Agilent MassHunter Quantitative Analysis B.06.00 software. The optimized method was validated for linearity, sensitivity, recovery and repeatability. The abundance of each N-glycan composition was relatively quantified based on the peak areas of their MRM chromatograms and expressed as a percentage of summed peak areas for total N-glycans within each IgG samples.

### Identification of sulfation sites using exoglycosidases

Four exoglycosidases, Sialidase C, *β*1–4 Galactosidase, *β*-N-Acetyl glucosaminidase, and *α*1–2,3 Mannosidase, were employed to hydrolyze the N-glycans and determine the monosaccharides linked to the sulfate groups.

Sialic acids were released by enzymatic digestion using Sialidase C. Briefly, N-glycans from 20 μg IgGs was reconstituted with 100 μl 50 mM NH_4_Ac (pH 5.0), and 5 μl Sialidase C^17^ (0.05 units) was added subsequently. The solution was incubated at 37 °C for 18 h, and the digestion was then terminated by heating the solution in boiling water for 5 min. The digestion was evaporated by speed vacuum and then resuspended into 40 μl H_2_O. After centrifugation at 14,000x *g* for 15 min, 30 μl of the supernatant was loaded into a vial insert with 1 μl of the acidic N-glycan IS. For the blank samples, 5 μl H_2_O was added instead.

*β*1–4 Galactosidase was employed to digest *β*1–4-linked galactose. In a total reaction volume of 10 μl, N-glycans from 20 μg IgGs and 1 μl *β*1–4 Galactosidase (8 units) were incubated in a sodium citrate (50 mM, pH 6.0) and NaCl (100 mM) reaction buffer for 1 h at 37 °C. After being diluted to 40 μl, the solution was centrifuged at 14,000×*g* for 15 min, and 30 μl of the supernatant was loaded into a vial insert with 1 μl of the acidic N-glycan IS. For the blank samples, 1 μl H_2_O was used instead of *β*1–4 Galactosidase.

*β*-N-Acetyl glucosaminidase was used to cleave the *β*-N-Acetyl glucosamine residues from oligosaccharides. Briefly, N-glycans from 20 μg IgGs and 1 μl *β*-N-Acetyl glucosaminidase (4 units) were incubated in a sodium citrate BSA buffer (50 mM, pH 6.0) for 4 h at 37 °C in a total volume of 10 μl. After being diluted to 40 μl, the solution was centrifuged at 14,000x *g* for 15 min, and 30 μl of the supernatant was loaded into a vial insert with 1 μl of the acidic N-glycan IS. For the blank samples, 1 μl H_2_O replaced *β*-N-Acetyl glucosaminidase.

*α*1–2,3 Mannosidase was employed to digest the *α*1–2,3 mannose residues from oligosaccharides. Briefly, N-glycans from 20 μg IgGs and 1 μl *α*1–2,3 Mannosidase (32 units) were incubated in a sodium acetate (50 mM, pH 5.5) and CaCl_2_ (5 mM) BSA buffer for 1 h at 37 °C in a total volume of 10 μl. After being diluted to 40 μl, the solution was centrifuged at 14,000×*g* for 15 min, and 30 μl of the supernatant was loaded into a vial insert with 1 μl of the acidic N-glycan IS. For the blank samples, 1 μl H_2_O replaced *α*1–2,3 Mannosidase.

All of the samples in the exoglycosidase experiments were prepared in duplicate.

### Statistical analysis

Comparisons were performed with the Mann–Whitney test for non-parametric continuous variables and Fisher’s exact test for categorical variables. Continuous variables were presented as mean ± s.d. Statistical significances were calculated using GraphPad Prism 6 (GraphPad Software, La Jolla, CA, USA). A two-sided *P*-value of < 0.05 was considered statistically significant. The sample size was not predetermined by statistical method. No data points were excluded from the analysis.Support Vector Machine (SVM) approach in Weka (University of Waikato), the software widely used for data mining, was used to generate the classification model. Attribute selection was performed using correlation-based feature subset selection (CfsSubsetEval) to select value subsets that correlate highly with the class value and lowly with each other. The best-first search strategy was used to navigate attribute subsets in search space. The quality or performance of the predicted models were evaluated by using the receiver operating characteristic (ROC) curve, which is obtained by calculating the sensitivity and specificity of the test at every possible cut off point, and plotting sensitivity (the proportion of true positive results) against 1-specificity (the proportion of false positive results). The method of Youden index (*J*) was employed to identify optimal cutoff points based on sensitivity, specificity and the ROC curve^56^. *J* was defined as the maximum vertical distance between the ROC curve and the diagonal or chance line and was calculated as *J = *maximum {sensitivity + specificity − 1}. Using this measure, the cut off point on the ROC curve which corresponded to *J* at which (sensitivity + specificity − 1) was maximized, was taken to be the optimal cut off point. The prediction model was then measured for sensitivity by using Eq. (1) and specificity by using Eq. (2),

1 $${\rm{Sensitivity}} = {\rm{TP}}/\left( {{\rm{TP}} + {\rm{FN}}} \right),$$

2 $${\rm{Specificity}} = {\rm{TN}}/\left( {{\rm{TN}} + {\rm{FP}}} \right),$$

where true positives (TP) denote the correct classifications of positive examples, true negatives (TN) are the correct classification of negative examples, false positives (FP) denote the incorrect classification of negative examples into the positive class and false negatives (FN) represent the incorrect classification of positive examples into the negative class.

The area under the ROC curve (AUC) was a reflection of how good the biomarker was at distinguishing RA patients and healthy individuals. The AUC served as a single measure, independent of prevalence, that summarized the discriminative ability of a test across the full range of cutoffs. The closer the AUC to 1, the better the overall diagnostic performance of the biomarker, and the closer it to 0.5, the poorer the test. All individual potential N-glycan biomarkers were further paired as different combinations and analyzed by the same strategy. Moreover, all potential single N-glycan biomarkers and combinations were applied in the prediction of RF-negative patients (*n* = 19) and ACPA-negative patients (*n* = 15), the prediction accuracy (%) was calculated as ((total number − mispredicted number) / total number × 100%).

For verification, the same classification models based on the identified individual or combined N-glycan biomarkers, together with their corresponding optimal cut off points generated from the training data sets, were applied to a validation set (187 RA patients and 84 healthy individuals). The prediction accuracy of RF-negative patients (*n* = 97) and ACPA-negative patients (*n* = 55) in the validation set were calculated by the same methods as that used in the training set.

### Data availability

The data that support the findings of the study are available from the corresponding author upon reasonable request.

## Electronic supplementary material

Supplementary information

Supplementary Data 1

Supplementary Data 2

Supplementary Data 3

Supplementary Data 4

Supplementary Data 5
