# Supplementary material for: A method to identify trace sulfated IgG N-glycans as biomarkers for rheumatoid arthritis
Source: Nat Commun. 2017 Sep 20;8:631. doi: 10.1038/s41467-017-00662-w (PMC5606999; doi:10.1038/s41467-017-00662-w)
Supplement: Supplementary file 1 — Supplementary information [file 41467_2017_662_MOESM1_ESM.pdf]

Description of Supplementary Files

File Name: Supplementary Information

Description: Supplementary figures, supplementary tables, supplementary methods

File Name: Supplementary Data 1

Description: Identification of acidic N-glycans released from the IgG of pooled human serum by using TiO2-PGC chip LC coupled with TOF MS

File Name: Supplementary Data 2

Description: Identification of neutral N-glycans released from the IgG of pooled human serum by using TiO2-PGC chip LC coupled with TOF MS

File Name: Supplementary Data 3

Description: MRM parameters for the quantification of N-glycans

File Name: Supplementary Data 4

Description: Quantitative profiling of N-glycans released from human serum IgGs

File Name: Supplementary Data 5

Description: Sensitivity and specificity of all combined N-glycan biomarkers for the classification of RA patients and healthy subjects

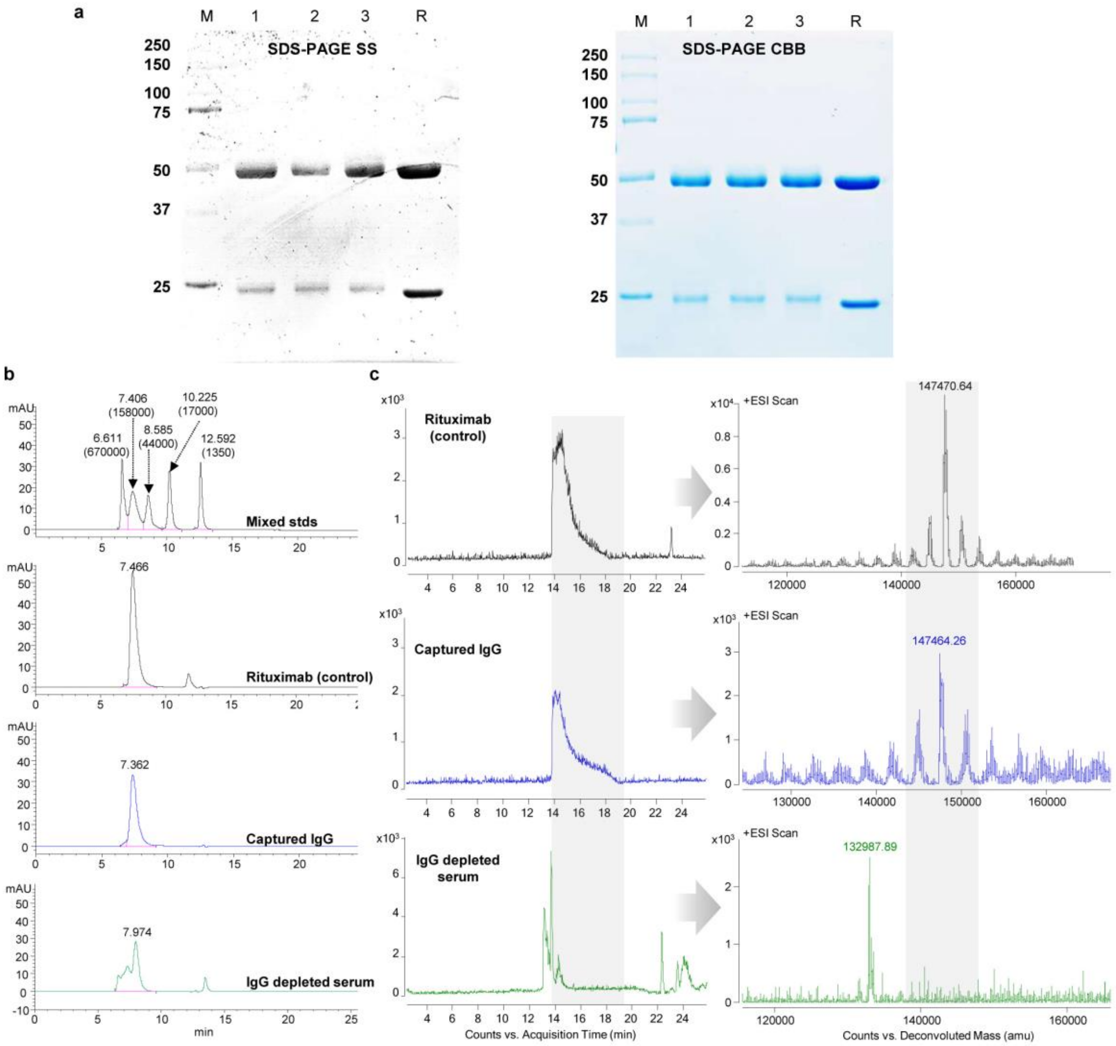

Supplementary Figure 1 | Purity of IgGs captured from pooled human serum. (**a**) SDS-PAGE of the serum IgG captured by using Protein A for 1-3 times (lane 1-3). Rituximab (lane "R") was employed as a reference. Left panel, silver staining (SS); right panel, Coomassie Brilliant Blue (CBB) staining. (**b**) HPLC-UV chromatograms of IgG. The samples were separated on an SEC column (Bio-Sil SEC 125-5, 300 x 7.8 mm, 5 μm). The mass of the captured IgG was calculated as approximately 158,000 based on the retention time and log (mass) of the mixed standards. (**c**) UHPLC-TOF MS chromatograms of the captured IgG and deconvoluted mass spectrum corresponding to the peak. The samples were separated on a C_8 UHPLC column (Kinetex 1.7 μm C_8 100 Å , Phenomenex).

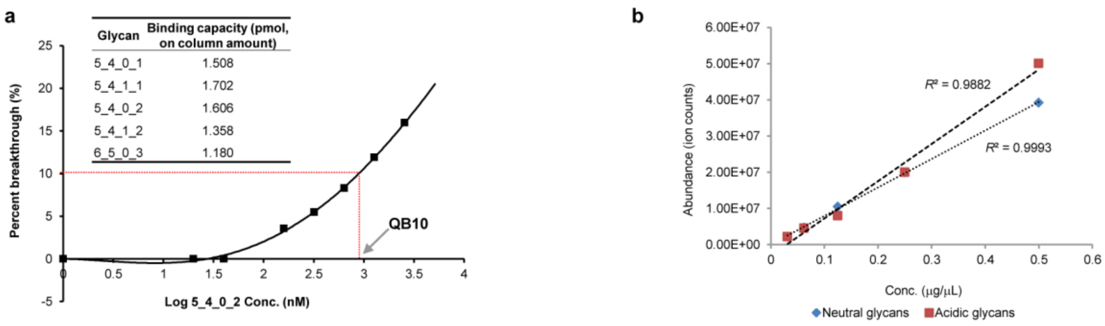

Supplementary Figure 2 | Acidic N-glycans binding capacity of the TiO_2-PGC chip. (**a**) The acidic N-glycans binding capacities were estimated using a breakthrough experiment. The binding capacities of five acidic N-glycan standards (defined as the on-column amount, pmol) are shown in the table inserted. (**b**) Under the optimized conditions, both neutral and acidic N-glycans obtained from 0.03-0.5 µg of IgG can be quantitatively eluted.

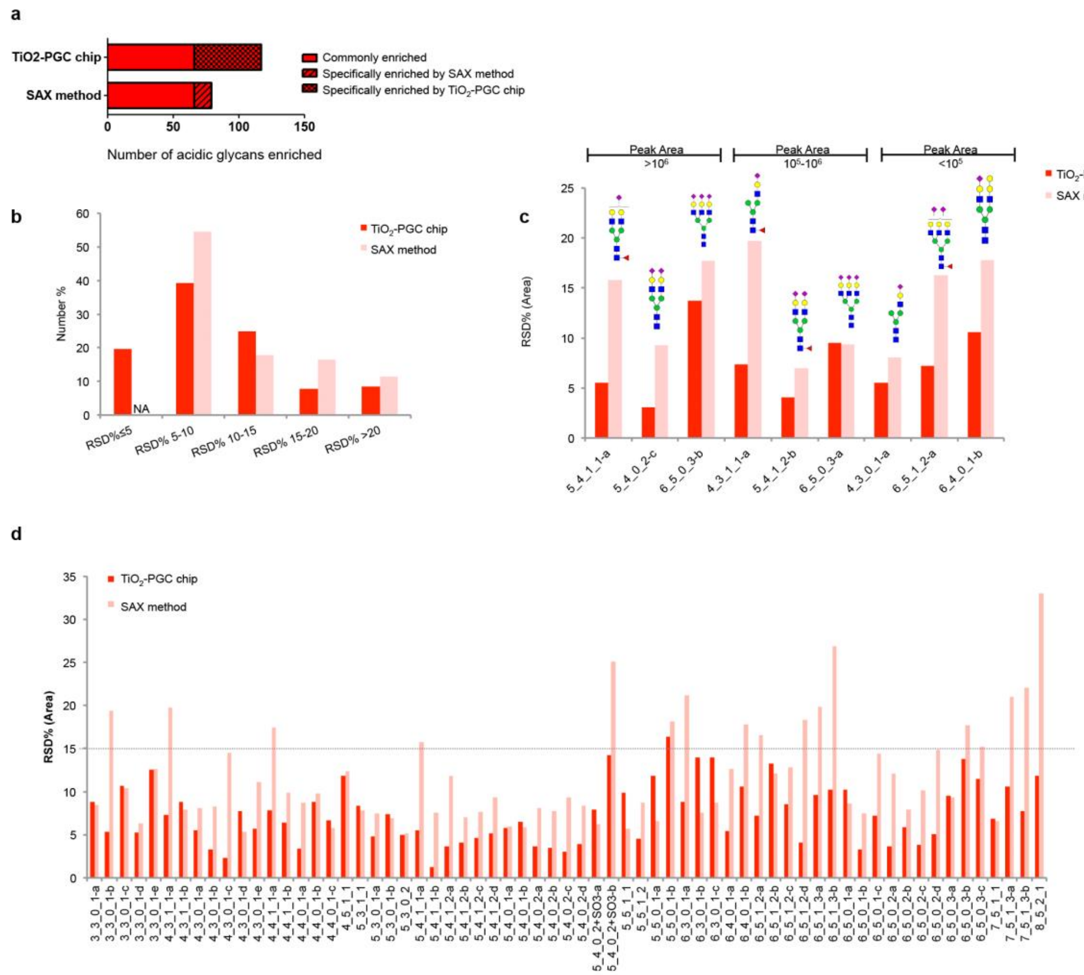

Supplementary Figure 3 | Comparison of the acidic N-glycans enrichment performance of the TiO_2-PGC chip and offline SAX. (**a**) Enrichment coverage of the TiO_2-PGC chip and SAX as represented by the number of acidic N-glycans enriched using the respective methods. (**b**) The overall reproducibility of the TiO_2-PGC chip and offline SAX, n = 6. The data are represented as the RSD% distribution of 117 acidic N-glycans enriched using the TiO_2-PGC chip and 79 N-glycans enriched using SAX, n = 6. The number % on the Y axis denotes the percentage of species with RSD% within a defined range as indicated on the X axis. (**c**) The enrichment reproducibility of 9 randomly selected acidic N-glycans whose signal intensities spanned the dynamic range observed in real biological samples using the TiO_2-PGC chip and SAX, n = 6. (**d**) Comparison of the enrichment reproducibility for individual acidic N-glycans using the TiO_2-PGC chip and offline SAX. The data are represented as the RSD% of the common acidic N-glycans enriched using both methods, n = 6. A total of 64 acidic N-glycans were enriched with an RSD within 15% using the TiO_2-PGC chip, whereas only 48 acidic N-glycans were enriched with an RSD less than 15% using SAX.

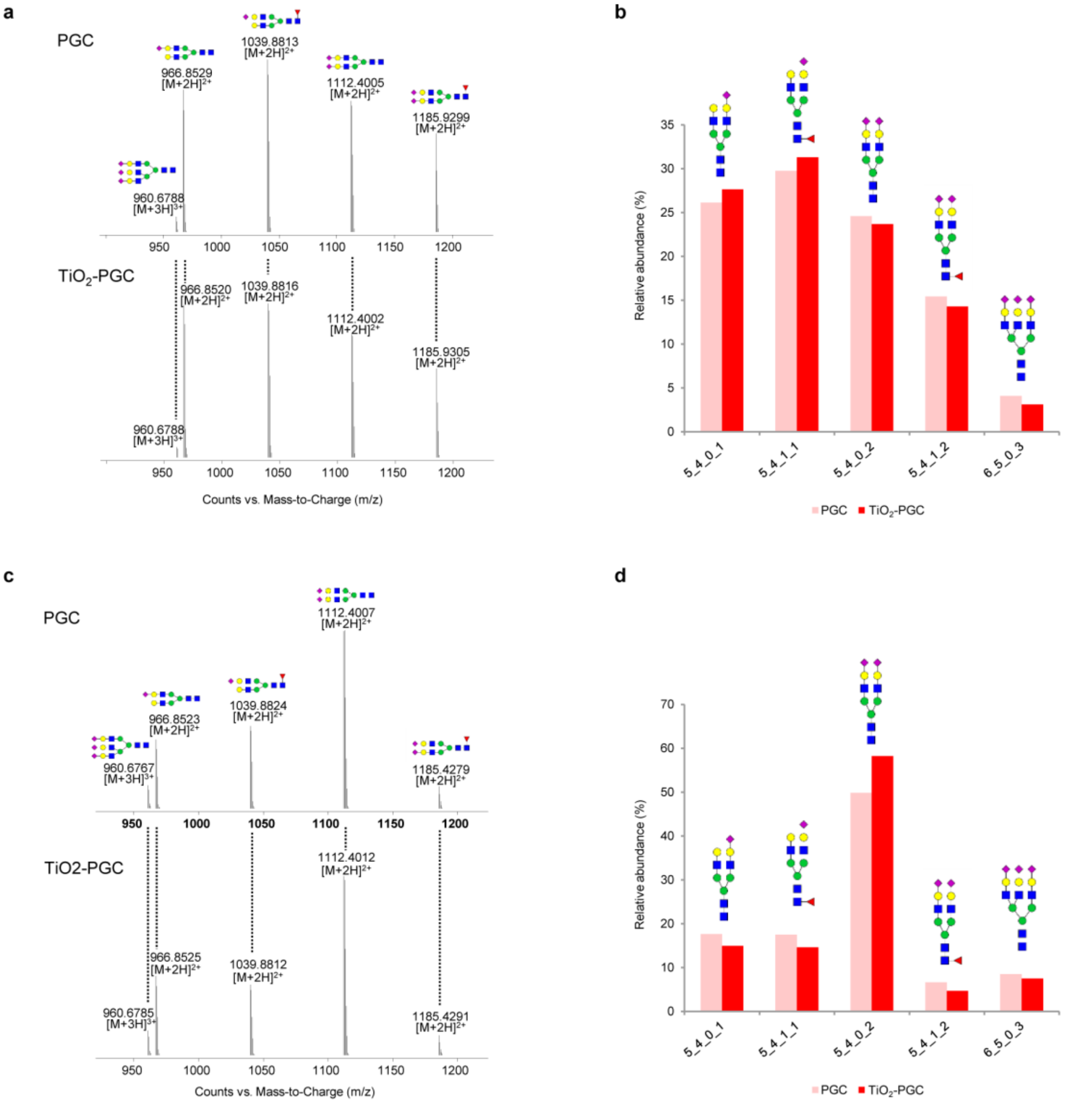

Supplementary Figure 4 | Reserved profile of acidic N-glycans after on-chip enrichment. The influence of on-chip enrichment on the profile of acidic N-glycans was examined using a set of acidic N-glycan standards and a complex acidic N-glycan mixture prepared from the total N-glycans released from human serum IgGs using the offline SAX method to ensure that “natural” and “real” acidic N-glycan profiles could be obtained after on-chip enrichment. (**a**, **c**) Full scan quadrupole time-of-flight mass spectra of 5 acidic N-glycan standards and a complex acidic N-glycan mixture analyzed using a routine PGC chip (without enrichment) and the TiO_2-PGC chip (with enrichment by TiO_2), respectively. (**b**, **d**) The relative abundances of all these species are conserved after enrichment by TiO_2.

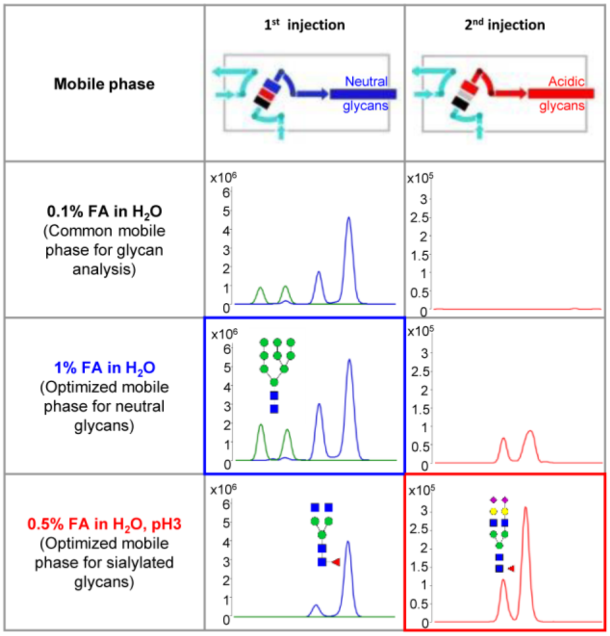

Supplementary Figure 5 | Substantially improved detection of both neutral and acidic N-glycans using optimized dual mobile phases.

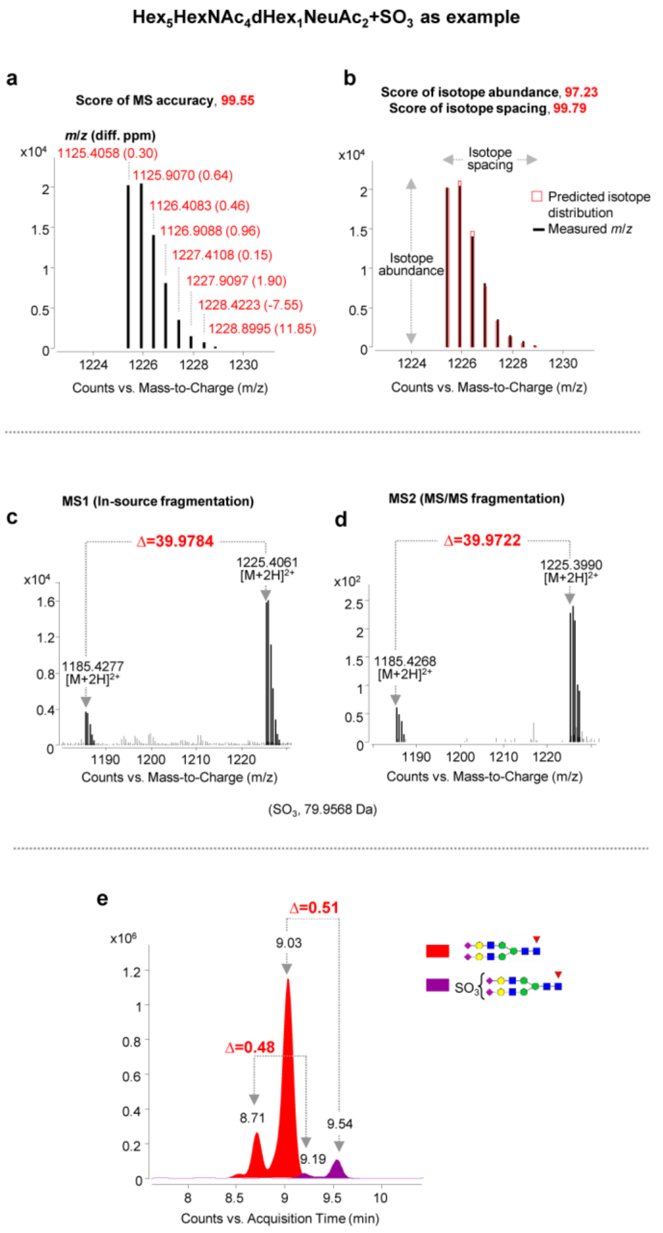

Supplementary Figure 6 | Characterization of sulfated N-glycans based on accurate mass. (**a**) MS accuracy, (**b**) isotope abundance and isotope spacing, (**c**) in-source fragmentation, (**d**) MS/MS fragmentation and (**e**) retention time of $Hex_5HexNAc_4dHex_1NeuAc_2$+$SO_3$.

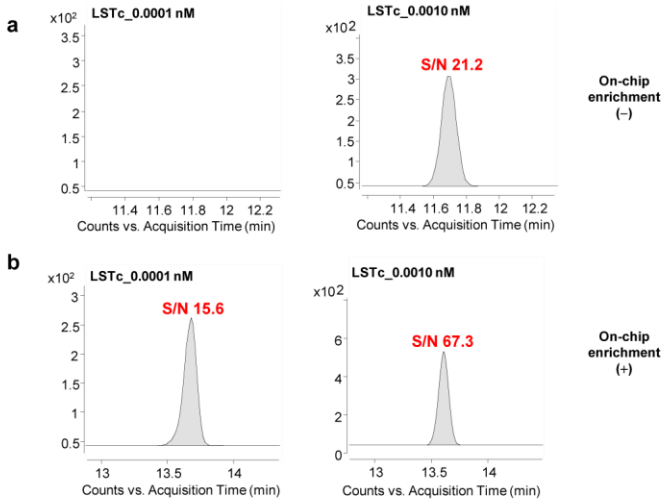

Supplementary Figure 7 | Enhanced detection sensitivity of acidic N-glycans after on-chip enrichment. Comparison of the MRM signal of the internal acidic O-glycan standard (LSTc) spiked into a complex neutral N-glycan mixture derived from the total N-glycans of serum IgG with (**b**) and without (**a**) on-chip enrichment.

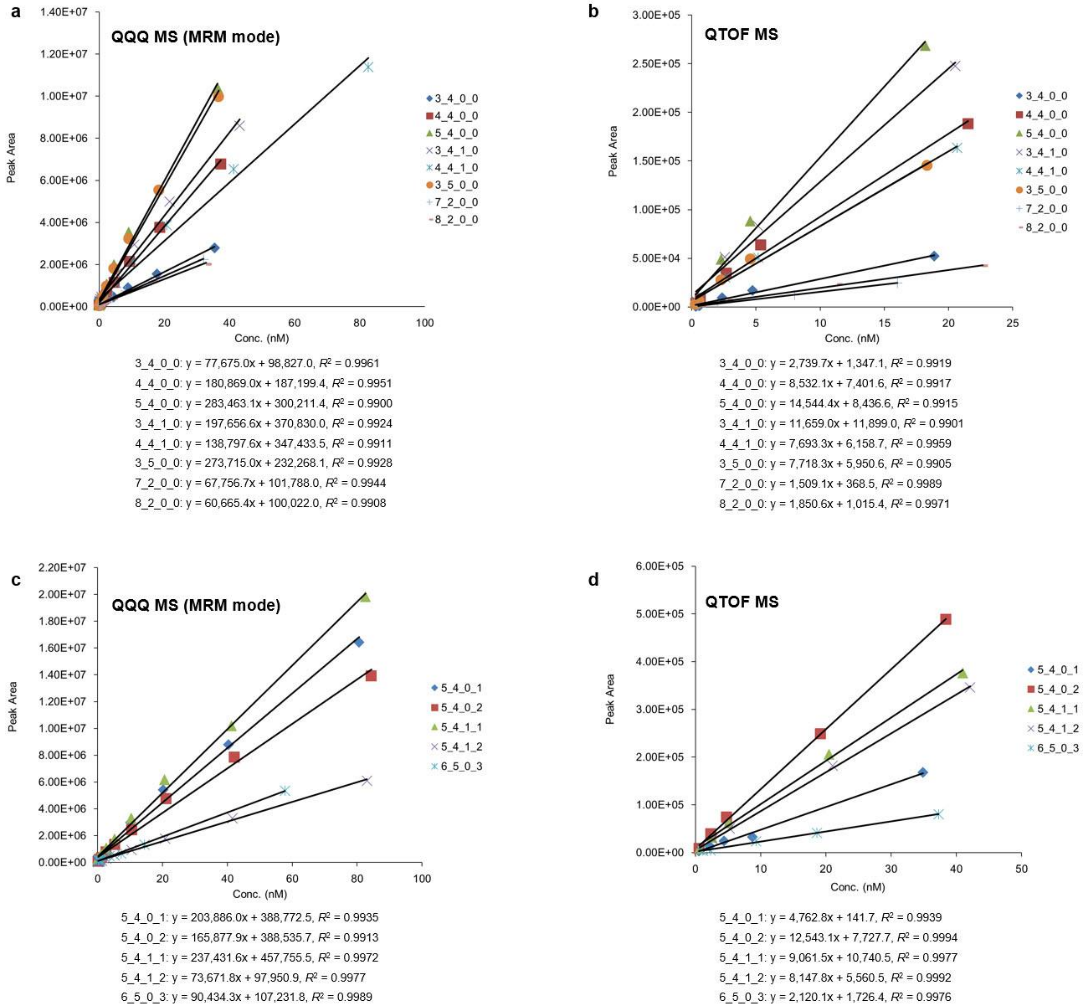

Supplementary Figure 8 | Linearity of 8 neutral and 5 acidic N-glycan standards measured on QQQ MS in the MRM mode and Q-TOF MS. Variation of neutral N-glycans (**a**, **b**) and acidic N-glycans (**c**, **d**) response factors (slope of the calibration curve, RF) measured via QQQ MS (maximum difference is 5-fold for neutral N-glycans and 3-fold for acidic N-glycans) is relatively smaller than that measured via Q-TOF MS (maximum difference is about 10-fold for neutral N-glycans and 6-fold for acidic N-glycans).

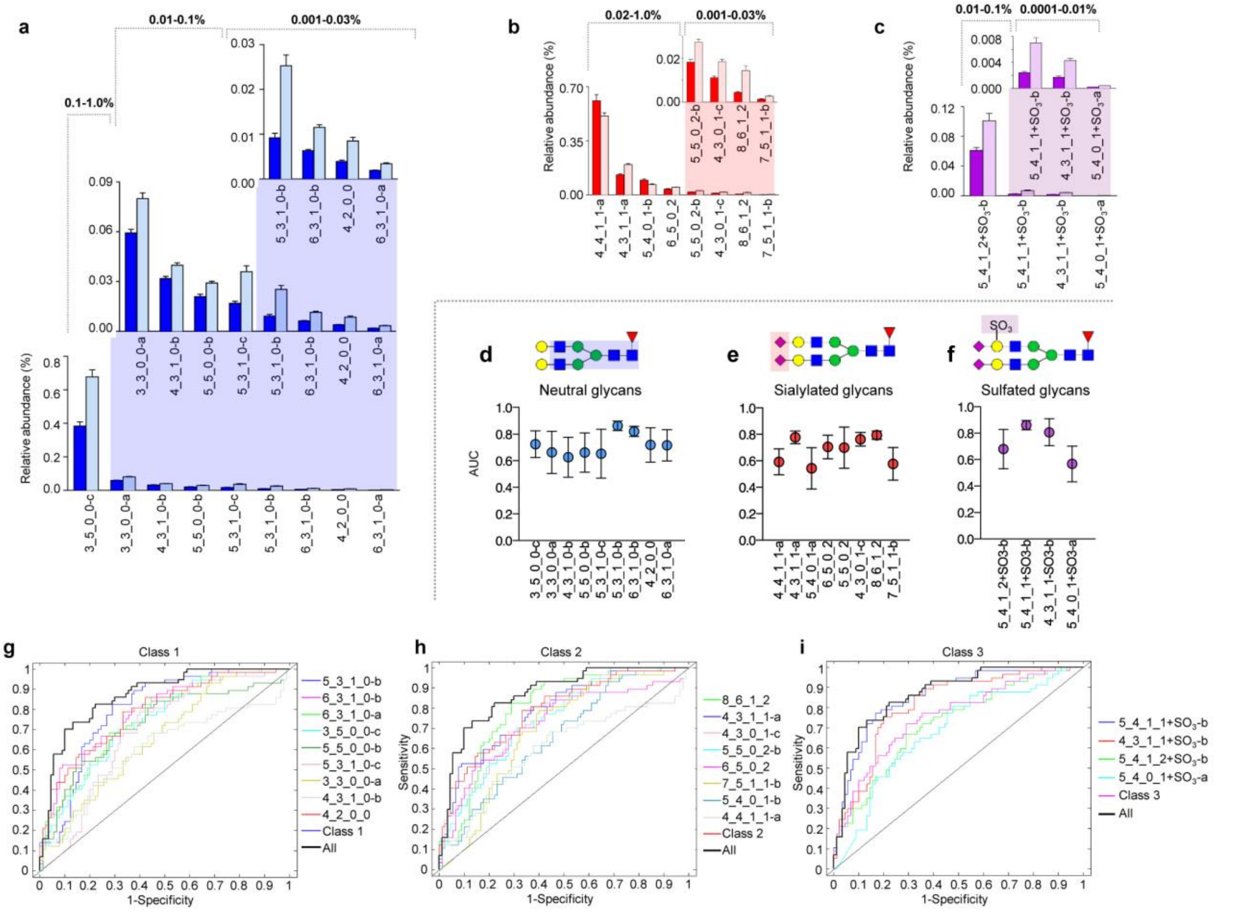

Supplementary Figure 9 | N-Glycan biomarkers of RA (Training set) (**a-c**) Relative abundances of 9 neutral, 8 sialylated and 4 sulfated N-glycan biomarkers on the serum IgGs of healthy subjects (dark column, *n* = 57) and RA patients (light column, *n* = 90), data are shown as mean ± s.d.. (**d-f**) AUC values for the ROC analysis of the potential N-glycan biomarkers, data are shown as mean ± s.d.. (**g-i**) ROC curves of neutral, sialylated and sulfated N-glycan biomarkers. In each of the plot, ROC curve of individual N-glycan biomarkers (with the legend of the N-glycan name), combined biomarkers of neutral N-glycans (with the legend of “class 1”), acidic N-glycans (with the legend of “class 2”) and sulfated N-glycan (with the legend of “class 3”) and all biomarkers (with the legend of “all”) were given.

Supplementary Table 1 | Comparison of the limit of quantitation (LOQ) of neutral and acidic N-glycan standards by using TOF MS and QQQ MS in MRM mode.

Name	Name	LOQ_QTOF (fmol)	LOQ_QQQ (fmol)
$Hex_5HexNAc_4NeuAc_1$	5_4_0_1	0.5441	0.016
$Hex_5HexNAc_4dHex_1NeuAc_1$	5_4_1_1	0.6389	0.008
$Hex_5HexNAc_4NeuAc_2$	5_4_0_2	0.5989	0.017
$Hex_5HexNAc_4dHex_1NeuAc_2$	5_4_1_2	0.6568	0.008
$Hex_6HexNAc_5NeuAc_3$	6_5_0_3	0.1453	0.003
$Hex_4HexNAc_4$	4_4_0_0	0.6717	0.015
$Hex_3HexNAc_4dHex_1$	3_4_1_0	0.6406	0.017
$Hex_4HexNAc_4dHex_1$	4_4_1_0	0.645	0.008
$Hex_3HexNAc_5dHex_1$	3_5_1_0	0.5623	0.007
$Hex_3HexNAc_6$	3_6_0_0	0.6791	0.007
$Hex_8HexNAc_2$	8_2_0_0	0.7073	0.007
$Hex_3HexNAc_1NeuAc_1$	LSTc	0.001	0.0001

Supplementary Table 2 | Linearity of neutral and acidic N-glycan standards by using TOF MS and QQQ MS in MRM mode.

Name	Abbreviation	QQQ				Q - TOF			
		Calibration equations	Linear range (nM)	R^2	n[a]	Calibration equations	Linear range (nM)	R^2	n[a]
$Hex_5HexNAc_4NeuAc_1$	5_4_0_1	y = 203,886.0x + 388,772.5	0.08 - 80.59	0.9935	11	y = 4,762.8x + 141.7	0.54 - 34.88	0.9939	5
$Hex_5HexNAc_4NeuAc_2$	5_4_0_2	y = 165,877.9x + 388,535.7	0.16 - 84.39	0.9913	10	y = 12,543.1x + 7,727.7	0.60 - 38.39	0.9994	5
$Hex_5HexNAc_4dHex_1NeuAc_1$	5_4_1_1	y = 237,431.6x + 457,755.5	0.08 - 82.57	0.9972	11	y = 9,061.5x + 10,740.5	0.64 - 40.96	0.9977	5
$Hex_5HexNAc_4dHex_1NeuAc_2$	5_4_1_2	y = 73,671.8x + 97,950.9	0.08 - 83.00	0.9977	11	y = 8,147.8x + 5,560.5	0.66 - 42.10	0.9992	5
$Hex_6HexNAc_5NeuAc_3$	6_5_0_3	y = 90,434.3x + 107,231.8	0.11 - 57.74	0.9989	9	y = 2,120.1x + 1,726.4	1.16 - 37.26	0.9976	5
$Hex_3HexNAc_4$	3_4_0_0	y = 77,675.0x + 98,827.0	0.07 - 35.46	0.9961	10	y = 2,739.7x + 1,347.1	0.29 - 18.89	0.9919	5
$Hex_4HexNAc_4$	4_4_0_0	y = 180,869.0x + 187,199.4	0.07 - 37.38	0.9951	10	y = 8,532.1x + 7,401.6	0.34 - 21.53	0.9917	5
$Hex_3HexNAc_4dHex_1$	3_4_1_0	y = 197,656.6x + 370,830.0	0.04 - 43.16	0.9924	11	y = 11,659.0x + 11,899.0	0.32 - 20.53	0.9901	5
$Hex_4HexNAc_4dHex_1$	4_4_1_0	y = 138,797.6x + 347,433.5	0.04 - 82.65	0.9911	12	y = 7,693.3x + 6,158.7	0.32 - 20.67	0.9959	5
$Hex_5HexNAc_4$	5_4_0_0	y = 283,463.1x + 300,211.4	0.07 - 36.34	0.9900	9	y = 14,544.4x + 8,436.6	0.28 - 18.17	0.9915	5
$Hex_3HexNAc_5$	3_5_0_0	y = 273,715.0x + 232,268.1	0.07 - 36.68	0.9928	10	y = 7,718.3x + 5,950.6	0.29 - 18.34	0.9905	5
$Hex_7HexNAc_2$	7_2_0_0	y = 67,756.7x + 101,788.0	0.12 - 32.05	0.9944	9	y = 1,509.1x + 368.5	0.50 - 16.02	0.9989	4
$Hex_8HexNAc_2$	8_2_0_0	y = 60,665.4x + 100,022.0	0.12 - 32.86	0.9908	9	y = 1,850.6x + 1,015.4	0.71 - 22.66	0.9971	4

[a] The point number of spiked calibration solutions used for linearity testing.

Supplementary Table 3 | The on-chip enrichment recovery rate (%) of acidic N-glycans (*n* = 3).

Name	Abbreviation	Acidic N-glycan standards [a]		Acidic N-glycan standards spiked into neutral N-glycan mixture of IgGs [a,b]	
		1.56 nM	12.5 nM	1.56 nM	12.5 nM
$Hex_5HexNAc_4NeuAc_1$	5_4_0_1	96.33 (17.42%)	90.73 (5.67%)	84.07 (12.90%)	105.93 (4.60%)
$Hex_5HexNAc_4dHex_1NeuAc_1$	5_4_1_1	92.07 (19.82%)	97.29 (9.14%)	83.70 (7.23%)	113.22 (2.33%)
$Hex_5HexNAc_4NeuAc_2$	5_4_0_2	105.39 (14.68%)	85.72 (13.88%)	112.30 (9.74%)	111.75 (6.97%)
$Hex_5HexNAc_4dHex_1NeuAc_2$	5_4_1_2	83.19 (4.84%)	81.27 (10.15%)	82.09 (13.99 %)	105.64 (15.09%)
$Hex_6HexNAc_5NeuAc_3$	6_5_0_3	92.76 (7.81%)	100.01 (18.93%)	89.89 (8.57%)	99.23 (18.62%)

[a] the number in the bracket denotes RSD of triplet measurements.
[b] neutral N-glycan mixture of IgGs was prepared from the total N-glycans released from the pooled human serum IgGs by using offline SAX method.

Supplementary Table 4 | Clinical features of AS patients and healthy subjects.

Clinical features	AS patients (n = 34)	Healthy subjects (n = 27)	P-value[#]
Age, years	37.9 ± 13.8	35.2 ± 13.5	0.37
Gender (female), %	17.6	33.3	0.23
Disease duration, years	10.7 ± 10.8	ND	
HLA-B27 positive, %	100	ND	
ESR, mm h^{-1}	27.9 ± 31.5	ND	
CRP, mg l^{-1}	2.1 ± 2.5	ND	

Values are the means ± s.d..

ND, not determined; HLA-B27, human leukocyte antigen B27; ESR, erythrocyte sedimentation rate; CRP, C-reactive protein

[#]*P*-values were calculated by the non-parametric Mann-Whitney test for age and by the Fisher's exact test for gender, two-sided.

Supplementary Table 5 | Clinical features of OA patients and healthy subjects

Clinical features	Knee OA patients (n = 26)	Healthy subjects (n = 45)	P-value[#]
Age, years	63.1 ± 17.1	59.3 ± 11.6	0.17
Gender (female), %	73.1	66.7	0.61
Disease duration, years	2.6 ± 4.5	ND	
ESR, mm h^{-1}	26.5 ± 23.9	ND	
CRP, mg l^{-1}	1.1 ± 2.5	ND	

Values are the means ± s.d..

ND, not determined; ESR, erythrocyte sedimentation rate; CRP, C-reactive protein

[#]*P*-values were calculated by the non-parametric Mann-Whitney test for age and by the Fisher's exact test for gender, two-sided.

Supplementary Methods

Materials and reagents. All N-glycan standards, including neutral N-glycans ($Hex_3HexNAc_4$, $Hex_4HexNAc_4$, $Hex_5HexNAc_4$, $Hex_3HexNAc_4dHex_1$, $Hex_4HexNAc_4dHex_1$, $Hex_5HexNAc_4dHex_1$, $Hex_3HexNAc_5$, $Hex_6HexNAc_5$, $Hex_3HexNAc_5dHex_1$, $Hex_5HexNAc_5dHex_1$, $Hex_3HexNAc_6$, and $Hex_7HexNAc_6$), acidic N-glycans ($Hex_5HexNAc_4NeuAc_1$, $Hex_5HexNAc_4NeuAc_2$, $Hex_5HexNAc_4dHex_1NeuAc_1$, $Hex_5HexNAc_4dHex_1NeuAc_2$, and $Hex_6HexNAc_5NeuAc_3$), high mannose N-glycans ($Hex_5HexNAc_2$, $Hex_6HexNAc_2$, $Hex_7HexNAc_2$, and $Hex_8HexNAc_2$), and an acidic O-glycan (LSTc), were purchased from Prozyme (Hayward, CA, USA). rProtein A Sepharose™ 4 Fast Flow (90 µm) was obtained from GE Healthcare (Uppsala, Sweden). PNGase F (500,000 units/mL) was purchased from New England BioLabs, Inc. (Beverly, MA, USA). The dye reagent concentrate for the protein assay was purchased from Bio-Rad (Hercules, CA, USA). Recombinant human N-acetylglucosamine-6-sulfatase (NG6S) was purchased from Novoprotein (Summit, NJ, USA), and galactose-6-sulfatase (GALNS) was purchased from Abnova. Sialidase C was purchased from Glyko Biomedical Ltd., and other exoglycosidases, including β1-4 galactosidase, β-N-acetyl glucosaminidase and α1-2,3 mannosidase, were purchased from New England BioLabs Inc. Multiscreen Solvinert Filter plates (96 wells, 0.45 µm, hydrophilic PTFE) and Amicon Ultra-0.5 100K centrifuge filter devices were purchased from Millipore (Merck Millipore, County Cork, Ireland). V-bottom 96-well collection plates and Sep-Pak C_{18} cartridges were purchased from GE Healthcare and Waters (Milford, MA, USA), respectively. SAX Ultra-Micro SpinColumns were purchased from Harvard Apparatus (Holliston, MA, USA), and HyperSep Hypercrab PGC cartridges were purchased from Thermo Fisher Scientific (Pittsburgh, PA, USA). Acetonitrile and methanol (both LC-MS grade) were purchased from Avantor (Center Valley, PA, USA), and LC-MS-grade FA, acetic acid and ammonia were purchased from Sigma-Aldrich (St. Louis, MO, USA). All other chemicals were of analytical reagent grade. Distilled water was prepared using a Milli-Q system (Millipore Ltd., Watford, UK).

Preparation of standard solutions. N-glycan standards were individually reconstituted in distilled water to yield stock solutions at a concentration of 100 µg ml^{-1}. The resulting individual stock solutions were then mixed and diluted to generate a series of working solutions (0.003-400 nM) for standard curve construction and the assessment of sensitivity and repeatability. Five

acidic N-glycan standards were mixed to prepare an additional stock solution of mixed acidic N-glycan standards, which was further diluted to 2 different concentrations (1.56 nM and 12.5 nM) to evaluate the on-chip enrichment recovery rate. For the binding capacity assessment, each acidic N-glycan was diluted individually to concentrations ranging from 10 to 5120 nM. All of the stock solutions were stored at -20°C before use, and all of the working solutions were freshly prepared.

Purity of the captured IgGs. Purity of the captured IgGs was examined by using SDS-PAGE and SEC method. For SDS-PAGE, analysis by Image J was performed to verify the purity of IgG. Both Silver Staining (SS) and Coomassie Brilliant Blue (CBB) staining demonstrated that the purity of captured IgGs was **greater than 99%.** In SEC method, purity defined as the percentage of peak area of IgGs relative to the sum of peak area of all proteins detected was calculated to be 97.3 %.

Binding capacity of acidic N-glycans on the TiO_2-PGC chip. The acidic N-glycans binding capacity of the TiO_2-PGC chip was evaluated using a breakthrough experiment. Serial concentrations of the individual acidic N-glycan standards were loaded onto and subsequently eluted from the enrichment column of the TiO_2-PGC chip. The amount of acidic N-glycan eluted in the loading and elution steps was determined using on-line detection. The overloaded fraction was collected from the waste tube and concentrated for analysis. The breakthrough percentage was calculated using the following formula: (signal in loading step + signal in overloaded fraction) / total signal × 100%. The breakthrough curve was constructed using the percentage of breakthrough against the N-glycan concentration, and the binding capacity was estimated as the amount of binding that occurred before the breakthrough percentage reached 10% (QB_{10}).

Offline enrichment of acidic N-glycans using SAX spin columns. SAX spin columns were preconditioned using 3 bed volumes of 0.05% FA aqueous solution 3 times. The total N-glycans released from serum IgG were resuspended in a 0.05% FA aqueous solution before passing through the preconditioned SAX spin column. The spin columns were then washed twice with 3 bed volumes of 0.05% FA, and the bound acidic N-glycans were eluted twice using 3 bed volumes of 1 M NaCl. The eluate was desalted using a PGC cartridge column and dried by speed vacuum. In the PGC desalting procedure, the column was preconditioned with 5 bed volumes each of ACN and 0.1% FA. The sample was loaded using gravity settling and then washed twice

with 1 bed volume of 0.1% FA. The bound acidic N-glycans were eluted twice using 1 bed volume of 80% ACN (pH 7.0, adjusted with 0.5% FA). The unbound neutral N-glycan fraction was treated as a complex neutral N-glycan mixture for subsequent validation experiments.

Comparison of the enrichment performance of the TiO_2-PGC chip and offline SAX for acidic N-glycans. The total N-glycans of the serum IgG were analyzed using the TiO_2-PGC chip coupled to a Q-TOF MS as described above, whereas the bound acidic N-glycans eluted from the SAX spin columns were analyzed using a PGC chip coupled to a Q-TOF MS. The number and pattern of acidic N-glycans obtained using both methods were compared to evaluate the enrichment of acidic N-glycans. Additionally, 6 replicates of the analysis under both methods were performed to assess the reproducibility of the TiO_2-PGC chip and offline SAX in terms of relative standard deviation (RSD%).

HPLC conditions for PGC chip. The PGC chip consisted of a 75 µm × 150 mm analytical column and a 75 µm × 9 mm enrichment column, and both were packed with 5 µm PGC as the stationary phase. A flow rate of 3 µl min^{-1} of 0.1% FA in water was used for sample loading with a 2 µl injection volume. The mobile phase for the nanopump consisted of 0.5% FA in water adjusted to pH 3 with an ammonia solution (A) and 1% FA in ACN (B) for acidic N-glycan analysis; for neutral N-glycans, the mobile phase consisted of 1% FA in water (A) and ACN (B). The flow rate was 0.5 µl min^{-1}, and the gradient was as follows: 5% B for 6 min, 5 to 60% B over 10 min, and 80% B for 3 min. An equilibrium time of 18 min was set before each injection.

Method validation. *Calibration curves.* A linear regression equation, $y = ax + b$, was defined to correlate the peak area to the N-glycan concentration. The linearity was verified using correlation coefficients (R^2), and the slope (a) of the standard curve represented the MS response factors of each N-glycan. The linear range and response factors of each N-glycan standard measured on QQQ MS in the MRM mode and Q-TOF MS were compared.

On-chip enrichment recovery. Two concentrations of an acidic N-glycan standard mixture (1.56 nM and 12.5 nM) and the same concentration of an acidic N-glycan standard mixture spiked into a complex neutral N-glycan mixture were analyzed using the TiO_2-PGC chip in the forward (with enrichment) and backward flush modes (without enrichment), respectively. The signal of each acidic N-glycan in both flush modes was measured in 6 replicates using QQQ MS in the

MRM mode. The recovery rate was calculated using the following formula: signal in forward flush mode / signal in backward flush mode × 100%.

Sensitivity. The limits of quantification (LOQs) were determined using the response at a S/N of 10. Using QQQ MS in the MRM mode and Q-TOF MS, the sensitivity of each N-glycan standard versus an internal acidic O-glycan standard (LSTc) was compared relative to LOQ. The acidic N-glycan sensitivity with and without on-chip enrichment was also compared according to the MRM signal of the LSTc, which was spiked into a complex neutral N-glycan mixture derived from the total N-glycans of serum IgG.

Repeatability. Three concentrations of a total N-glycan standard mixture (0.19 nM, 1.56 nM and 12.5 nM) were analyzed using the TiO_2-PGC chip and QQQ MS in the MRM mode for 6 replicates. The repeatability of the TiO_2-PGC chip was compared with that of the PGC chip in terms of RSD%.
